# The Maize (*Zea mays* L.) *AUXIN/INDOLE-3-ACETIC ACID* Gene Family: Phylogeny, Synteny, and Unique Root-Type and Tissue-Specific Expression Patterns during Development

**DOI:** 10.1371/journal.pone.0078859

**Published:** 2013-11-01

**Authors:** Yvonne Ludwig, Yanxiang Zhang, Frank Hochholdinger

**Affiliations:** Crop Functional Genomics, Institute of Crop Science and Resource Conservation, Rheinische-Friedrich-Wilhelms Universität, Bonn, Germany; Universidad Miguel Hernández de Elche, Spain

## Abstract

The plant hormone auxin plays a key role in the coordination of many aspects of growth and development. *AUXIN/INDOLE-3-ACETIC ACID* (*Aux/IAA*) genes encode instable primary auxin responsive regulators of plant development that display a protein structure with four characteristic domains. In the present study, a comprehensive analysis of the 34 members of the maize *Aux/IAA* gene family was performed. Phylogenetic reconstructions revealed two classes of Aux/IAA proteins that can be distinguished by alterations in their domain III. Seven pairs of paralogous maize Aux/IAA proteins were discovered. Comprehensive root-type and tissue-specific expression profiling revealed unique expression patterns of the diverse members of the gene family. Remarkably, five of seven pairs of paralogous genes displayed highly correlated expression patterns in roots. All but one (*ZmIAA23*) tested maize *Aux/IAA* genes were auxin inducible, displaying two types of auxin induction within three hours of treatment. Moreover, 51 of 55 (93%) differential *Aux*/*IAA* expression patterns between different root-types followed the expression tendency: crown roots > seminal roots > primary roots > lateral roots. This pattern might imply root-type-specific regulation of *Aux/IAA* transcript abundance. In summary, the detailed analysis of the maize *Aux/IAA* gene family provides novel insights in the evolution and developmental regulation and thus the function of these genes in different root-types and tissues.

## Introduction

The phytohormone auxin plays an essential role in plant growth and development. Auxin controls many aspects of plant morphology and physiology [1,2] such as apical dominance, tropisms and the differentiation of vascular tissues [3]. Moreover, auxin affects division, elongation, and differentiation of cells [3,4]. On the molecular level, auxin controls gene expression [5,6] and membrane functions [7]. Several auxin-responsive genes have been identified and characterized including the GRETCHEN HAGEN 3 (GH3), SMALL AUXIN-UP RNA (SAUR), and *AUXIN/INDOLE-3-ACETIC ACID* (*Aux/IAA*) gene families [8]. In etiolated tissue of soybean (*Glycine max*), *Aux/IAA* genes were initially identified because of their fast induction by auxin [9]. Subsequently, 29 members of the *Arabidopsis thaliana Aux/IAA* gene family [10], 26 members of the tomato and sorghum *Aux/IAA* families [11-13], and 31 *Aux/IAA* genes were identified in rice [14] and maize [15], respectively. *Aux/IAA* genes are unique to plants and have not been identified in bacteria, animals or fungi [16]. In *Arabidopsis thaliana*, not all *Aux/IAA* genes are inducible by auxin. For instance, *AtIAA17* and *AtIAA28* display only a minor or no response to exogenous auxin-treatment [17-19]. Members of the *Aux/IAA* gene family encode short-lived nuclear proteins which consist of four characteristic domains [2,20,21]. Aux/IAA proteins function as transcriptional repressors of downstream auxin-regulated genes [22,23] *via* a short conserved leucine repeat motif (LxLxLx) in domain I. Domain II, with the conserved degron-sequence GWPPV, is responsible for the stability of Aux/IAA proteins [23]. The interaction of domain II with the F-box protein TIR1 leads to a rapid degradation of Aux/IAA proteins [24]. Point mutations in the degron sequence or deletions of this sequence [25] stabilize Aux/IAA proteins which can result in specific developmental phenotypes [10,16]. Domains III and IV of Aux/IAA proteins homo and heterodimerize with other Aux/IAA proteins or auxin response factors (ARFs) [26,27]. Moreover, interaction of these domains with domain III and IV of ARFs control expression of downstream auxin responsive genes [26,27]. ARF proteins interact with auxin-responsive *cis*-elements (AuxRE) in the promoter of downstream auxin responsive genes [27-29]. Finally, Aux/IAA proteins contain a nuclear localization signal (NLS) which targets these proteins to the nucleus. Typically, Aux/IAA proteins contain two NLS, one is separated into two parts including the short sequence KR between domain I and II, and a six amino acid sequence in domain II. The second NLS is located at the end of domain IV at the carboxy-terminus [30].

To date only one member of the maize Aux/IAA family has been characterized in detail. RUM1/ZmIAA10 displays characteristics of a canonical Aux/IAA protein including nuclear localization, short half life time and the possibility to interact with ARF proteins [25]. Deletion of 26 amino acids including the degron sequence in the mutant *rum1-R* resulted in a root-specific phenotype blocking the initiation of embryonic seminal and postembryonic lateral roots in the primary root [31].

In the present study, phylogenetic and syntenic relations of the *Aux/IAA* gene family members in maize were determined and a comprehensive expression and correlation analysis of different root and shoot tissues of all maize *Aux/IAA* genes during development was performed, unveiling root-type and tissue-specific expression patterns that might help to understand the diverse functions of these genes in root development. 

## Materials and Methods

### Plant material, growth conditions, and hormone treatment

Seeds of the maize inbred line B73 were sterilized with 6% sodium hypochlorite for 10 min and rinsed in distilled water. Subsequently, seeds were rolled up in germination paper (Anchor paper, www.anchorpaper.com) [32] and transferred to 10 l buckets filled with ~3 l distilled water. Germinating seedlings were incubated at 28 °C with a 16 h light and 8 h dark cycle. Five-day-old maize seedlings were treated with 5 mM α-naphthyl acetic acid (αNAA) working solution for 3 h. The differentiation zone of two to three primary roots per biological replicate was harvested each hour. Coleoptiles were harvested from seedlings grown for four days at 28 °C in the dark. Seedling samples were harvested at different developmental stages and were immediately frozen in liquid nitrogen and stored at -80 °C until RNA isolation. 

### The Aux/IAA gene family, novel members, phylogeny, and synteny

In the initial version of the maize reference genome sequence B73 RefGen_v1 [33] 31 *Aux/IAA* genes were predicted among 32,540 protein-encoding genes [15]. To obtain a comprehensive overview of the maize *Aux/IAA* gene family, the 31 previously identified maize *Aux/IAA* genes were used as query sequences for blast (http://blast.ncbi.nlm.nih.gov/Blast.cgi) searches of the maize filtered gene set based on genome assembly version AGPv2 (http://www.maizegdb.org/), containing 39,656 high confidence genes. 

The protein sequences of the maize *Aux/IAA* genes were retrieved from MaizeGDB (http://maizegdb.org/). Moreover, the previously identified rice Aux/IAA protein sequences [15] were retrieved from the Rice Genome Annotation Project (http://rice.plantbiology.msu.edu/index.shtml), and the published sorghum sequences [34] were extracted from Gramene (http://www.gramene.org/). The four conserved domains in the maize *Aux/IAA* gene family were determined by multiple alignments with ClustalW (http://www.clustal.org/). Synteny of the maize sequences was determined with Comparative Genomics software (CoGe, http://genomevolution.org/CoGe/; [35]) and association with maize subgenomes 1 and 2 were based on [36]. 

Phylogenetic analyses comparing maize, rice, and sorghum Aux/IAA protein sequences were conducted using the neighbor-joining algorithm in MEGA5 [37] considering 1,000 replications with bootstrap analyses. 

### RNA isolation and cDNA synthesis

Frozen maize shoot and root tissues were ground and approximately 100 mg per biological replicate were used for total RNA extraction via the RNeasy Plant Mini Kit (Qiagen, http://www.qiagen.com/). Subsequently, RNA was treated with RNase-free DNAse I (Fermentas, http://www.thermoscientificbio.com/fermentas/). To exclude the possibility of DNA contamination, the RNA samples were tested via PCR with oligonucleotides for maize *actin 1* (AY104722) that bind to exon sequences that flank an intron. For cDNA synthesis 500 ng of total RNA was subjected to the qScript cDNA Synthesis Kit protocol (Quanta BioScience, http://www.quantabio.com/). For each root-type and tissue, five biological replicates were analyzed while auxin induction was tested in three biological replicates. Three technical replicates were measured for each sample.

### Quantitative real-time-PCR

Expression of maize *Aux/IAA* genes was determined by quantitative real-time-PCR in a Bio-Rad CFX 384™ Real-Time System (http://www.bio-rad.de) using gene-specific oligonucleotides (Table S1). The oligonucleotides were designed by Primer3Plus (http://www.bioinformatics.nl/cgi-bin/primer3plus/primer3plus.cgi) and checked with NetPrimer (PREMIER Biosoft, http://www.premierbiosoft.com) software. Each reaction contained 4 µl MESA Blue qPCR™ Mastermix Plus for SYBR Assay no ROX (Eurogentec, http://www.eurogentec.com), 1 µl cDNA sample and 250 nM gene-specific oligonucleotide primers to a final volume of 8 µl. The primer efficiency of each oligonucleotide was calculated using the following dilution series: 1, 1/2, 1/4, 1/8, 1/16, 1/32, 1/64, and 1/128. The relative expression levels of the transcripts were calculated with reference to the housekeeping gene *myosin* (Genbank AC: 486090G09.x1). Differential gene expression was determined by a two sided Student´s t-test.

### Identification of expression patterns for auxin inducible Aux/IAA genes

Two types of expression patterns, A and B, were identified for auxin-inducible *Aux/IAA* genes in maize within 3 hours of auxin induction. For all genes auxin induction at three time points t_1_, t_2_, t_3_ was compared to the control at t_0_. Pattern A is characterized by a significantly increased expression at one time point compared to the control t_o_. In pattern A increased expression remained at later time points significantly above the control. In pattern B, expression at t_1_ or t_2_ was significantly increased compared to t_0_, while expression at t_2_ or t_3_ was significantly decreased compared to t_1_ or t_2_, respectively.

## Results

### The maize Aux/IAA family consists of 34 members

To obtain a comprehensive overview of the maize *Aux/IAA* gene family, the previously identified 31 maize *Aux/IAA* sequences [15] were used as query to screen version 2 of the maize filtered gene set comprising 39,656 high confidence genes (ZmB73_5b_FGS; http://www.maizegdb.org/). As a result, three novel maize *Aux/IAA* genes which display the canonical four domain structure were discovered including *ZmIAA32* (GRMZM2G366373), *ZmIAA33* (GRMZM2G359924), and *ZmIAA34* (GRMZM2G031615) increasing the total number of maize *Aux/IAA* genes to 34 (Table 1). Aux/IAA domains were identified via a SMART (Simple Modular Architecture Research Tool; http://smart.embl-heidelberg.de/smart/set_mode.cgi) search. Proteins encoded by 29 *Aux/IAA* genes displayed all four domains. By contrast, in ZmIAA25 domains I and II are lacking and domains III and IV are incomplete. Moreover, domain II is absent in ZmIAA24 and ZmIAA26 whereas domain IV is missing in ZmIAA22 and ZmIAA31. Furthermore, in ZmIAA28 domain IV is split in two parts by the insertion of nine amino acids (Figure S1). A summary of the characteristics of the 34 maize *Aux/IAA* genes and the proteins encoded by them is provided in Table 1. The four Aux/IAA domains are highlighted in an alignment of the Aux/IAA protein sequences by ClustalW (Figure S1). The five ZmIAA proteins ZmIAA3, ZmIAA9, ZmIAA13, ZMIAA24, and ZmIAA26 displayed a modified LxLxPP instead of the predominant LxLxLP motif in domain I, whereas the 13 Aux/IAA protein sequences ZmIAA4, ZmIAA6, ZmIAA9, ZmIAA11, ZmIAA12, ZmIAA16, ZmIAA17, ZmIAA18, ZmIAA20, ZmIAA23, ZmIAA30, ZmIAA33, and ZmIAA34 displayed a variation in the conserved motif of domain III. 

**Table 1:** Characteristics of the maize *Aux/IAA* gene family.

***Name***	***AC maizeGDB***	***Maize chromo-some***	***Genome location***	***Strand***	***Protein length (aa)***	***Sub-genome 1***	***Sub-genome 2***	***Maize chromo-some with syntenic region***	***ZmIAA gene in syntenic region***	***Rice chromo-some with syntenic region***	***Sorghum chromo-some with syntenic region***
***ZmIAA1***	GRMZM2G079957_T2	1	170.535.129- 170.536.982	1	227	X		3	*ZmIAA8*	12	8
***ZmIAA2***	GRMZM2G159285_T1	1	275.031.309- 275.034.076	-1	237	X		5	*ZmIAA14*	3	1
***ZmIAA3***	GRMZM5G809195_T1	1	288.394.822- 288.396.335	-1	202	X		5	*ZmIAA13*	3	1
***ZmIAA4***	GRMZM2G104176_T1	3	7.117.449- 7.120.087	-1	229	X				1	3
***ZmIAA5***	GRMZM2G004696_T1	3	10.073.330- 10.076.684	1	220	X		8	*ZmIAA27*	1	3
***ZmIAA6***	GRMZM2G074742_T1	3	48.981.283- 48.983.063	1	198	X				1	3
***ZmIAA7***	GRMZM2G138268_T1	3	117.766.041- 117.770.646	-1	271		X			12	8
***ZmIAA8***	GRMZM2G167794_T1	3	118.064.610- 118.066.326	-1	230		X	1	*ZmIAA1*	12	8
***ZmIAA9***	GRMZM2G057067_T1	3	199.305.498- 199.307.986	-1	357	X				1	3
***ZmIAA10***	GRMZM2G037368_T1	3	209.094.740- 209.098.102	-1	269	X		8	*ZmIAA29*	1	3
***ZmIAA11***	GRMZM2G059544_T2	4	39.557.305- 39.558.529	-1	251		X			8	7
***ZmIAA12***	GRMZM2G142768_T1	4	171.373.109- 171.375.531	1	293		X			2	4
***ZmIAA13***	GRMZM2G152796_T1	5	4.200.507- 4.201.934	-1	181		X	1	*ZmIAA3*	3	1
***ZmIAA14***	GRMZM2G077356_T1	5	7.772.505- 7.775.451	1	228		X	1	*ZmIAA2*	3	1
***ZmIAA15***	GRMZM2G128421_T1	5	17.343.149- 17.344.582	1	224						
***ZmIAA16***	GRMZM2G121309_T1	5	151.712.395- 151.716.302	1	289	X				2	4
***ZmIAA17***	GRMZM2G030465_T1	5	214.411.995- 214.413.229	1	206	X				2	4
***ZmIAA18***	GRMZM2G000158_T4	6	79.914.748- 79.916.495	-1	197		X	9	*ZmIAA30*	6	10
***ZmIAA19***	GRMZM2G079200_T1	6	103.139.630- 103.141.327	-1	198		X			6	10
***ZmIAA20***	GRMZM5G864847_T1	6	130.004.758- 130.006.167	-1	234						
***ZmIAA21***	GRMZM2G147243_T2	6	133.196.856- 133.200.588	1	244	X		8	*ZmIAA28*	5	9
***ZmIAA22***	GRMZM2G141205_T1	6	146.505.719- 146.506.414	-1	231						
***ZmIAA23***	GRMZM2G074427_T2	6	160.166.708- 160.170.208	1	346	X				5	9
***ZmIAA24***	GRMZM2G149449_T1	7	9.392.913- 9.393.634	1	115						
***ZmIAA25***	GRMZM2G115357_T2	7	10.970.395- 10.971.721	-1	66	X				7	2
***ZmIAA26***	GRMZM2G048131_T1	7	142.773.908- 142.775.365	1	139	X				9	2
***ZmIAA27***	GRMZM2G130953_T2	8	18.318.828- 18.320.919	-1	186		X	3	*ZmIAA5*	1	3
***ZmIAA28***	GRMZM2G035465_T3	8	110.812.538- 110.816.166	-1	256		X	6	*ZmIAA21*	1	9
***ZmIAA29***	GRMZM2G163848_T5	8	150.560.417- 150.563.479	1	272		X	3	*ZmIAA10*	5	3
***ZmIAA30***	GRMZM2G001799_T1	9	16.249.556- 16.251.444	1	216	X		6	*ZmIAA18*	6	10
***ZmIAA31***	GRMZM2G134517_T1	10	134.255.159- 134.255.926	-1	255						
***ZmIAA32***	GRMZM2G366373_T2	1	253.302.210- 253.303.545	-1	226						
***ZmIAA33***	GRMZM2G359924_T1	8	108,568,427- 108,570,670	1	228						
***ZmIAA34***	GRMZM2G031615_T2	4	16,418,192- 16,426,336	1	355	X				11	5

#### Phylogeny and synteny of the maize Aux/IAA family

**Figure 1:**
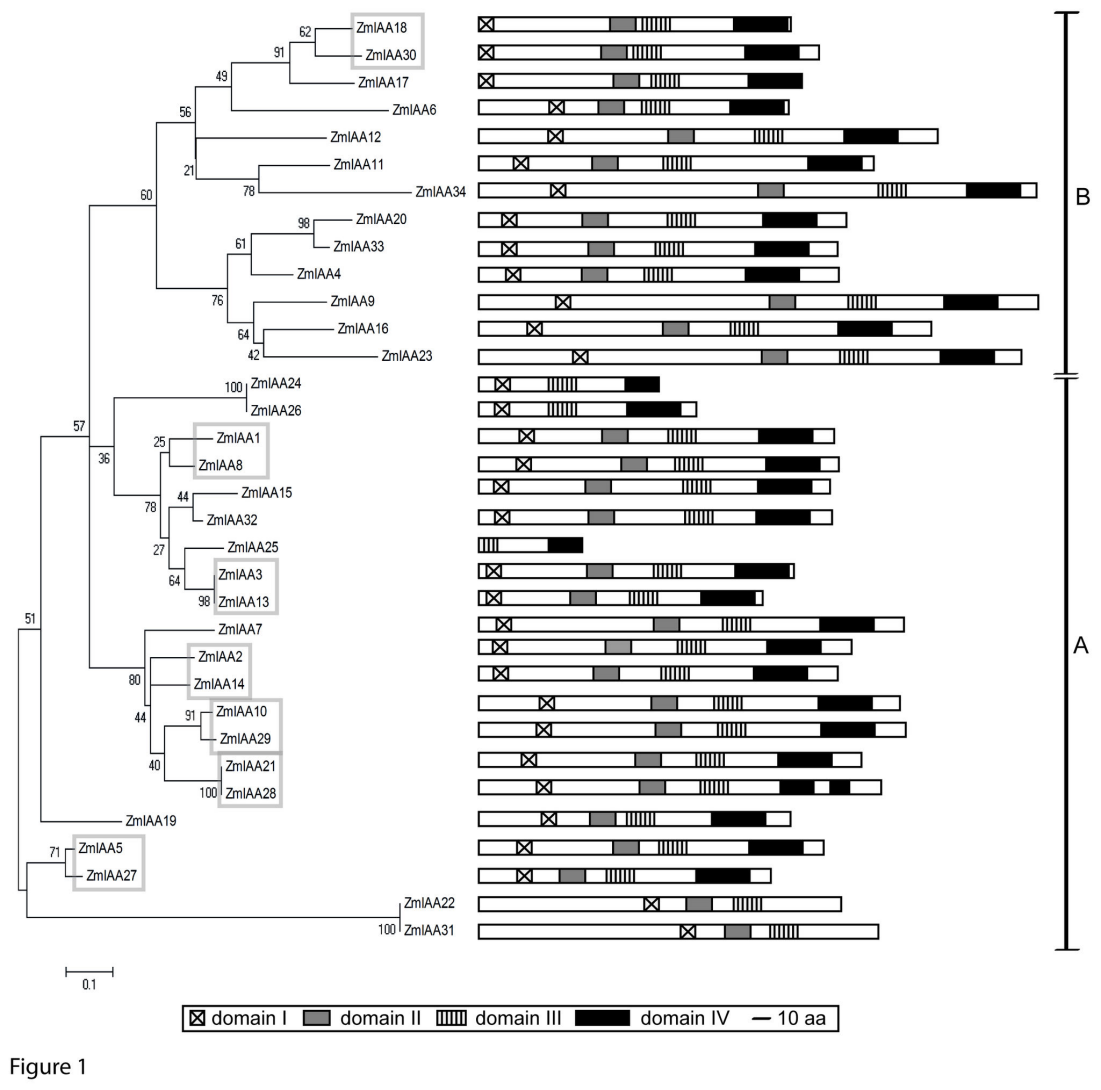
Unrooted phylogenetic tree and distribution of conserved domains in maize Aux/IAA proteins. The phylogenetic tree reveals two classes (A and B) of Aux/IAA proteins that differ in the sequence of domain III. The structure of the Aux/IAA proteins and the distribution of their domains are displayed to the right. The scale bar corresponds to 10 amino acids (aa). Paralogous *Aux/IAA* genes are encircled. The values associated to each branch are bootstrap percentages. The size bar indicates sequence divergence : 0.1 = 10%.

Phylogenetic reconstructions were based on the full-length sequences of the 34 maize Aux/IAA proteins. Two major groups of Aux/IAA proteins (class A and class B) were observed (Figure 1), which coincided with the alteration in the conserved motif in domain III (Figure S1).

Evolution and synteny of the maize *Aux/IAA* gene family was studied via CoGe (http://genomevolution.org/CoGe/; Table 1). In total, seven pairs of paralogous maize *Aux/IAA* genes were identified (Table 1, Figure 1). Moreover, for seven *Aux*/*IAA* genes (*ZmIAA15*, *ZmIAA20*, *ZmIAA22*, *ZmIAA24*, *ZmIAA31*, *ZmIAA32*, and *ZmIAA33*) no orthologs were found in rice and sorghum suggesting that these genes are the result of gene duplications after the separation of maize from rice and sorghum. 

Functional diversification of monocot maize, rice, and sorghum *Aux/IAA* genes was studied by a phylogenetic reconstruction of the Aux/IAA proteins of these species (Figure S2). 

### Expression of the Aux/IAA gene family during development

*Aux/IAA* gene expression was determined in embryonic primary and seminal roots and post-embryonic lateral and shoot-borne roots. Moreover, primary roots were surveyed at different developmental stages. Finally, different root tissues of the primary root including the meristematic and elongation zones and cortex and stele tissues of the differentiation zone were analyzed. Expression levels were determined by quantitative RT-PCR for 30 of 34 *Aux/IAA* genes. For the closely related genes *ZmIAA22*/*ZmIAA31* and *ZmIAA24*/*ZmIAA26* (Figure 1) no specific oligonucleotides were available that allowed to distinguish between them. Moreover, these four genes encode for Aux/IAA proteins that do not display the canonical four domain structure.

**Figure 2:**
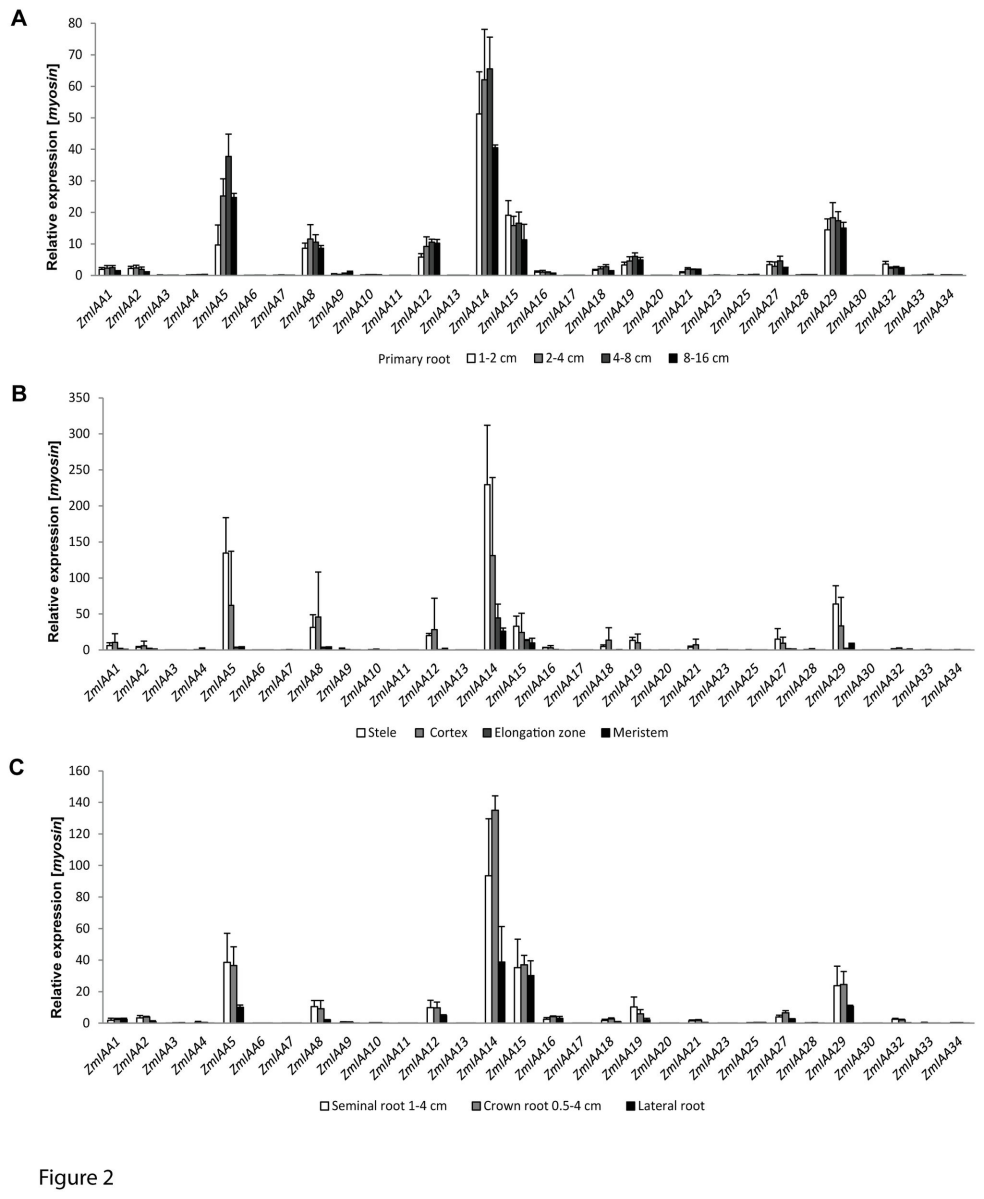
Expression of *Aux/IAA* genes during development in different root-types and tissues. Relative expression of 30 *Aux/IAA* genes was surveyed via qRT-PCR relative to *myosin* during primary root development (A), in different primary root tissues (B), and in seminal, crown and lateral roots (C).

Expression patterns largely differed between the different maize *Aux/IAA* genes (Figure 2). *ZmIAA5*, *ZmIAA8*, *ZmIAA12*, *ZmIAA14*, *ZmIAA15*, and *ZmIAA29* displayed the highest expression levels in primary roots (Figure 2A). *ZmIAA14* contributes ~40% of all *Aux/IAA* transcripts in 1-2 cm primary roots and ~35% in later developmental stages of the primary root. While *ZmIAA14* levels in young primary roots (up to the 4-8 cm class) are not significantly different, expression of this gene significantly decreases during the later developmental stages 4-8 cm and 8-16 cm (Figures S3 and S4). *ZmIAA5* displayed the second highest *Aux/IAA* expression in primary roots and provides 8% of the *Aux/IAA* transcripts in 1-2 cm primary roots and 20% in 8-16 cm primary roots. *ZmIAA5* displayed a significant increase in gene expression between 1-2 cm primary roots and 4-8 cm primary roots (Figures S3 and S4). As in *ZmIAA14*, expression of *ZmIAA5* decreases between 4-8 cm and 8-16 cm primary roots. 

These six *Aux/IAA* genes also displayed the highest expression in primary root tissues three days after germination (Figure 2B). Preferential expression in cortex and stele tissues compared to the meristematic and elongation zones was detected for 27 of 30 tested *Aux/IAA* genes (Figure S3). Only *ZmIAA11* and *ZmIAA13* displayed the highest expression in the root apical meristem while in *ZmIAA34* expression in the meristematic and elongation zones were slightly higher than in the stele. The six genes that displayed the highest expression in primary roots also displayed the highest expression in seminal, crown and lateral roots (Figure 2C). 

When analyzing differential gene expression of maize *Aux/IAA* genes between different root-types, a total 19 of genes (*ZmIAA1*, *ZmIAA2*, *ZmIAA4*, *ZmIAA5*, *ZmIAA8*, *ZmIAA13*, *ZmIAA14*, *ZmIAA15*, *ZmIAA16*, *ZmIAA18, ZmIAA20, ZmIAA21, ZmIAA23, ZmIAA25, ZmIAA27, ZmIAA28, ZmIAA30, ZmIAA33, ZmIAA34*) displayed significantly higher expression in crown roots compared to at least one stage of primary root development (Figures S3 and S4). In contrast, only *ZmIAA6* was significantly lower expressed in crown roots than in the three stages of primary root development (Figure S3 and S4). Moreover, four genes were preferentially expressed in crown versus lateral roots (*ZmIAA2*, *ZmIAA13*, *ZmIAA28*, *ZmIAA30*) and another four genes were preferentially expressed in crown versus seminal roots (*ZmIAA16*, *ZmIAA27*, *ZmIAA28*, *ZmIAA30*). Hence, among 27 genes differentially expressed between crown roots and other root-types 26 display a higher expression in crown roots. When comparing differential gene expression between primary, seminal and lateral roots eleven genes were differentially expressed between primary and lateral roots (*ZmIAA5*, *ZmIAA7*, *ZmIAA8*, *ZmIAA9*, *ZmIAA12*, *ZmIAA13*, *ZmIAA18*, *ZmIAA21*, *ZmIAA27*, *ZmIAA32, ZmIAA34*). All of these genes except *ZmIAA27* displayed a higher expression in primary versus lateral roots. Moreover, nine genes were differentially expressed between at least one stage of primary root development and seminal roots (*ZmIAA3*, *ZmIAA6*, *ZmIAA9*, *ZmIAA13*, *ZmIAA16*, *ZmIAA21*, *ZmIAA25*, *ZmIAA28*, *ZmIAA30*). Only *ZmIAA6* and *ZmIAA9* were preferentially expressed in primary roots, while the remaining seven genes were preferentially expressed in seminal roots. Finally, seven genes were preferentially expressed in seminal versus lateral roots (*ZmIAA7*, *ZmIAA8*, *ZmIAA10*, *ZmIAA13*, *ZmIAA20*, *ZmIAA21*, *ZmIAA34*). These differential expression patterns display a strong bias for expression levels between the different root-types, which applies to 51 of 55 (93%) of the pairwise differential expression patterns between different root-types observed in the present study. This pattern suggests that differentially expressed *Aux/IAA* genes follow in most instances the following hierarchy of expression: crown roots > seminal roots > primary roots > lateral roots. 

To complement the expression profile of the *Aux/IAA* gene family, different shoot tissues of young maize seedlings were analyzed (Figure S3). The genes *ZmIAA6*, *ZmIAA9*, and *ZmIAA20* displayed a significantly higher expression in shoot tissues than in any root tissue. In all three instances the genes were highly expressed in the mesocotyl. Moreover, for some *Aux/IAA* genes expression in the coleoptile was higher than in some of the root tissues (Figure S3). Such a preferential expression was however not observed for leaves and coleoptilar node. 

### Correlation of gene expression in different root-types and tissues of paralogous Aux/IAA gene pairs

**Figure 3:**
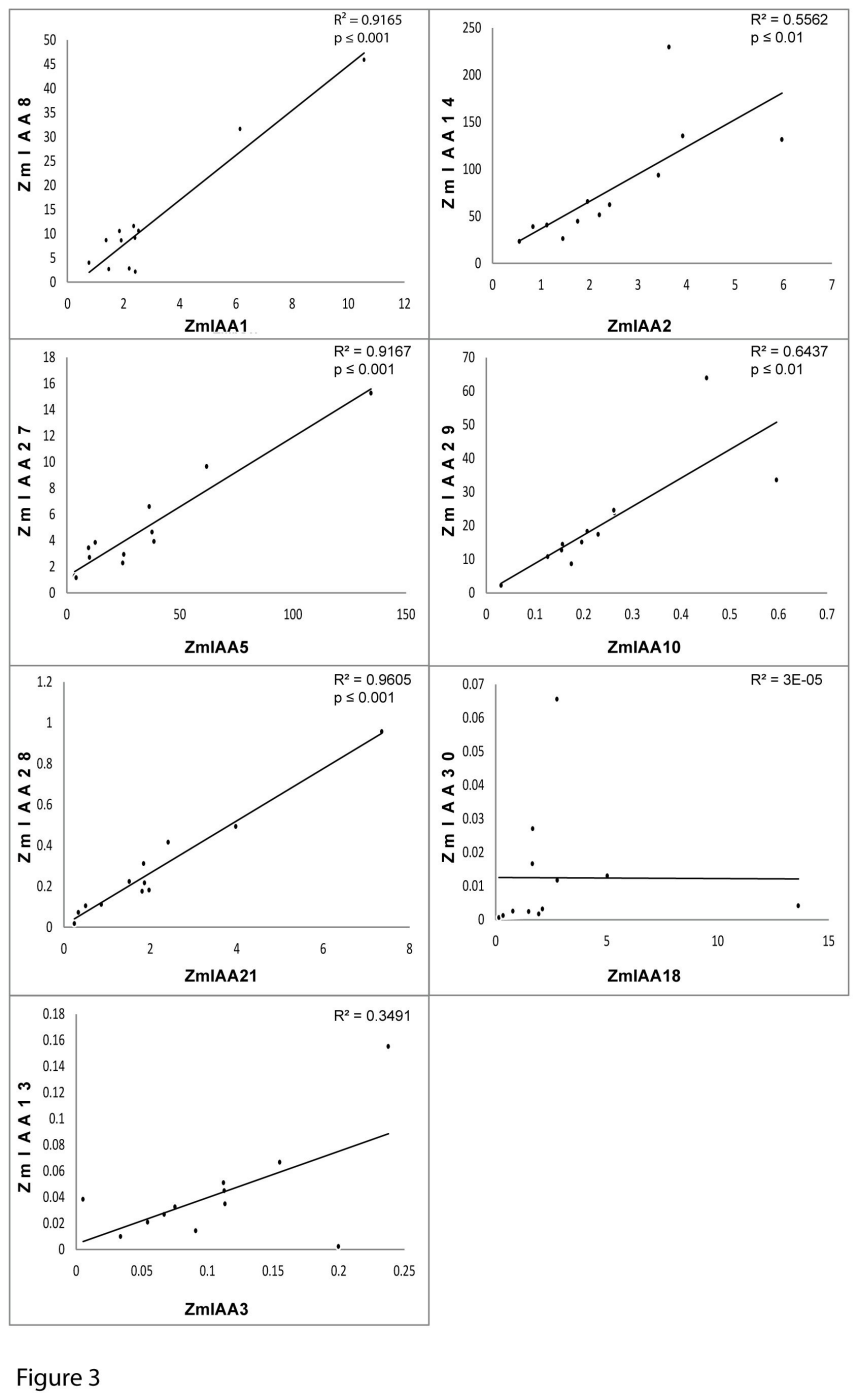
Correlation of gene expression of the seven paralogous *Aux/IAA* gene pairs in maize roots. Five of seven paralogous *Aux/IAA* pairs showed a significant correlation in their gene expression patterns in roots (coefficient of determination R^2^ >0.5; *p* ≤0.01). Only *ZmIAA30*/*ZmIAA18* and *ZmIAA13*/*ZmIAA3* did not display significant expression correlation in the tested root-types and tissues.

To compare expression patterns between the seven paralogous maize gene pairs in roots, coefficients of determination were calculated based on the gene expression data (Figure 3). This analysis revealed a strong linear correlation (*p* ≤0.01) for five of seven gene pairs up to R^2^ = 0.96 (*ZmIAA21*/*ZmIAA28*). Nevertheless, for all five pairs of paralogs one of the genes displayed on average a significantly higher expression level than the other one. The paralogous pairs *ZmIAA3*/*ZmIAA13* and *ZmIAA18*/*ZmIAA30* did not display any significant correlation with respect to their expression patterns in roots.

The paralogous *Aux/IAA* pairs *ZmIAA1*/*ZmIAA8*, *ZmIAA5*/*ZmIAA27*, *ZmIAA21*/*ZmIAA28*, and *ZmIAA3*/*ZmIAA13* revealed a higher expression level of the gene located in maize subgenome 1 compared to the paralog in subgenome 2. In the other three pairs *ZmIAA2*/*ZmIAA14*, *ZmIAA10/ZmIAA29*, and *ZmIAA18/ZmIAA30* the gene of subgenome 2 was preferentially expressed.

### Two types of auxin-induced Aux/IAA expression kinetics in maize

**Figure 4:**
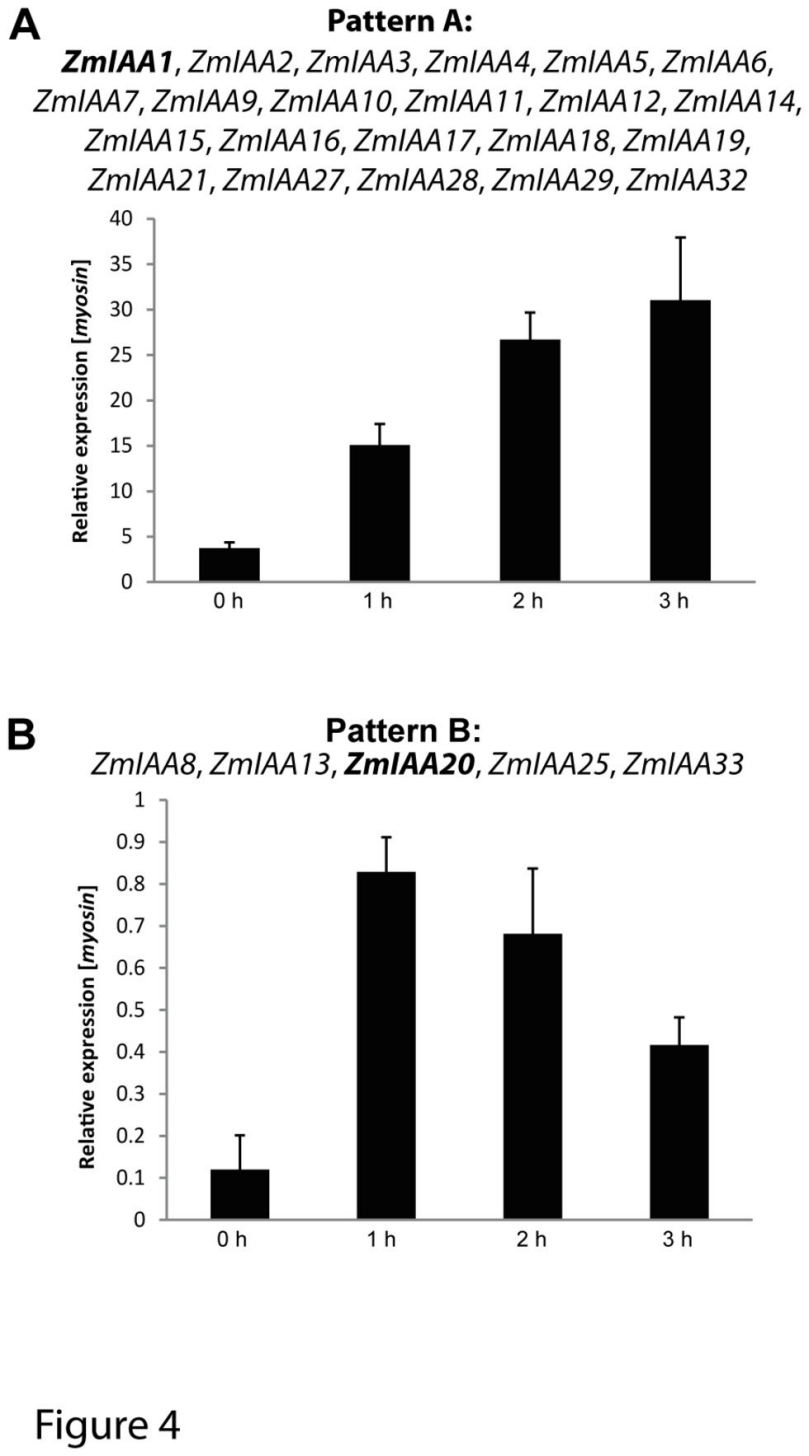
Auxin (αNAA) induction of maize *Aux/IAA* genes. Two patterns of auxin induction were identified by qRT-PCR in the differentiation zone of 5-day-old maize primary roots after 5 µM αNAA (α-Naphthalene Acetic Acid) treatment over three hours. Genes were either constitutively induced (A) or expression decreased relative to the initial induction (B). Each pattern is illustrated by one example (in bold) while other *Aux/IAA* genes that followed these induction patterns are listed. All tested *Aux/IAA* genes were αNAA inducible except *ZmIAA23*. A detailed account of the *Aux/IAA* induction results is provided in Figure S5.

Screening for *cis*–elements in the regulatory region 3 kb upstream of ATG revealed that 28 of 34 analyzed maize *Aux/IAA* promoters contain canonical auxin response elements (*AuxRE*) 5´ TGTCTC 3´ or its inverse complement sequence 5´ GAGACA 3´ (Table S2). Moreover, all 34 maize *Aux/IAA* promoters contain several multiple tandem copies of the *AuxRE* core sequence 5´ TGTC 3´ or 5´GACA 3´. The presence of *AuxRE* motifs and its derivatives suggests that these genes are regulated by auxin. Therefore, auxin induction of the maize *Aux/IAA* gene family was tested in the differentiation zone of young maize primary roots over a time period of 3 h by quantitative real-time PCR (Figure S5). Two distinct expression patterns were observed after auxin treatment (Figure 4). In pattern A at least one time point displayed significantly increased expression compared to t_0_ and expression at t_3_ was still significantly higher than at t_0_ (Figure 4A and Figure S4). Moreover, in pattern B expression at t_1_ or t_2_ was significantly increased compared to t_0_, whereas expression at t_2_ or t_3_ was significantly decreased compared to t_2_ or t_1_, respectively (Figure 4B and Figure S4). The majority (22 of 27) of maize *Aux/IAA* genes displayed pattern A (Figure 4A and Figure S4). Pattern B was observed for *ZmIAA8*, *ZmIAA13*, *ZmIAA20*, *ZmIAA25*, and *ZmIAA33* (Figure 4B and Figure S4). Moreover, *ZmIAA30* and *ZmIAA34* were below the detection limit to observe any expression. *ZmIAA23* was the only maize gene that was not induced by auxin (Figure S4). 

## Discussion

### Novel Aux/IAA genes and structural analyses of maize Aux/IAA family

*Aux/IAA* genes are plant-specific transcriptional regulators [16]. Initially, 31 maize *Aux/IAA* genes were discovered in the maize genome [15]. Improved annotation allowed for the identification of three novel *Aux/IAA* genes (*ZmIAA32*: GRMZM2G366373, *ZmIAA33*: GRMZM2G359924, *ZmIAA34*: GRMZM2G031615) in the present study, increasing the total number in maize to 34. Similarly, the rice genome contains 31 *Aux/IAA* genes [14] while sorghum harbors 26 *Aux/IAA* genes in its genome. 

Sequence analysis of the maize Aux/IAA protein family revealed that five maize Aux/IAA proteins do not contain all four domains characteristic of this protein family. Similarly, domain II which is required for the degradation of the Aux/IAA proteins is partially or totally missing in OsIAA4, OsIAA8, OsIAA27, OsIAA28, and OsIAA29 in rice [14] and in AtIAA20, AtIAA30, AtIAA31, AtIAA32, AtIAA33, and AtIAA34 in *Arabidopsis* [38]. Moreover, tomato Sl-IAA32 does not have domain II and Sl-IAA33 is lacking domains I and II [12]. 

Furthermore, in several genes modifications of characteristic amino acid sequences were observed. The LxLxLx motif in domain I is known to function as a repressor and is characteristic of flowering plants. In contrast, LxLxPP is typically found in mosses or in one of the three *Aux/IAA* genes of the vascular non-seed plant *S. moellendorffii* [39]. Nevertheless, the sequence LxLxPP was also observed in five maize Aux/IAA proteins (ZmIAA3, ZmIAA9, ZmIAA13, ZmIAA24, and ZmIAA26), while in *Arabidopsis* and in rice no LxLxPP motives were found. The vascular non-seed plant *S. moellendorffii* and flowering plants do not form monophyletic groups, suggesting that the motif was established independently in each lineage. In *Arabidopsis*, it was reported that specific point mutations in codons that encode the leucine residue in the domain I motif lead to a weaker repression of ARF-mediated transcription [23]. Although there is no evidence that LxLxPP functions as a repressor domain, it was proposed to be functionally important due to its wide distribution outside the flowering plants [39]. 

### Synteny and correlation in gene expression

The diversity and complexity of the *Aux/IAA* gene family in modern maize can be explained by gene and genome duplications and by gene loss because of partial fractionation. The maize genome was duplicated ~5-12 million years ago [40]. As a consequence, pairs of paralogous *Aux/IAA* genes emerged in maize. Today, seven pairs of paralogous *Aux/IAA* genes are retained in the maize genome. This implies that the maize *Aux/IAA* gene family was already diversified by gene duplications before this whole genome duplication event. Based on their synteny with sorghum, a species whose genome has not been duplicated, maize genes can be allocated to the maize subgenomes 1 or 2. The chromosomal regions with less gene loss were designated subgenome maize 1 [41]. A classical model of gene duplication assumes that one duplicated gene maintains the original function, while a second copy is lost, silenced or evolves a new function [42]. Hence, some of these gene pairs might have diversified by subfunctionalization or neofunctionalization [43]. Five of the seven paralogous *Aux/IAA* gene pairs displayed a significant correlation of their gene expression patterns in roots. Among those, only one pair (*ZmIAA21*/ZmIAA*28*) also showed similar expression patterns in shoot tissues. The correlation in gene expression for five of seven *Aux/IAA* gene pairs might also imply functional redundancy of these gene pairs. Nevertheless, even for these gene pairs functional diversification cannot be excluded because the average expression levels of these gene pairs are significantly different. Similarly, functional redundancy has been reported for *Arabidopsis* and rice *Aux/IAA* genes [17,39,44].

A subset of the duplicated genes has been lost since the ancient genome duplication by intrachromosomal recombination, silencing or null mutations [40], an ongoing process called fractionation [36]. In the maize *Aux/IAA* gene family, thirteen genes which can be assigned to either subgenome 1 or 2 but for which no paralog was retained are the result of fractionation. Finally, seven *Aux/IAA* genes were not assigned to any of the maize subgenomes. These genes likely emerged after the ancient maize genome duplication. Therefore, the *Aux/IAA* gene family in modern maize is the result of ancient gene duplications, a more recent whole genome duplication, partial fractionation, and modern gene duplications. 

### Expression profiling of Aux/IAA during development

In the present study, a systematic expression analysis of different maize root-types, tissues and developmental stages revealed root and tissue-specific expression patterns. The six maize *Aux/IAA* genes *ZmIAA5*, *ZmIAA8*, *ZmIAA12*, *ZmIAA14*, *ZmIAA15*, and *ZmIAA29* displayed an overall high expression in all tested embryonic and postembryonic roots. This might suggest that these *Aux/IAA* genes play a constitutive role during maize root development. Similarly, the rice genes *OsIAA5*, *OsIAA6*, and *OsIAA23* displayed the highest expression levels in roots [14]. Among those, *OsIAA5* is the ortholog of the maize paralogs *ZmIAA10/rum1* and *ZmIAA29*. Hence, both of the closely related genes *OsIAA5* and *ZmIAA29* display a very high expression in roots [14]. Similarly, *OsIAA23* and *ZmIAA5* which both display high expression levels in roots map to the same phylogenetic clade. However, while *ZmIAA14* displays the highest transcript level of all tested maize *Aux/IAA* genes, its rice ortholog *OsIAA13* displays only low expression in roots. Hence, while some rice and maize genes might have conserved their function in root development during evolution other members of the gene family might have not. Interestingly, none of ten cotton *Aux/IAA* genes displayed a major expression peak in roots [29].

Among the 30 maize *Aux/IAA* genes tested in the present study, only three genes displayed significantly higher expression in non-root tissue than in any of the tested root samples. In contrast, among the 31 rice *Aux/IAA* genes only *OsIAA6* and *OsIAA23* revealed their expression maximum in six day-old roots compared to shoot tissues. The maize ortholog of *OsIAA6* is *ZmIAA9*, which displayed its expression maximum in the mesocotyl. In contrast, *ZmIAA19*, which is the ortholog of the rice gene *OsIAA23*, displays higher expression levels in all root-types than in any of the analyzed shoot tissues. This supports the notion that even among the closely related maize and rice *Aux/IAA* gene families a functional diversification of the gene family members has occurred during evolution [14]. However, very different tissues and developmental stages were analyzed in these maize and rice studies which makes them difficult to compare.

Thus far, mutant analyses of maize *Aux/IAA* genes revealed a developmental phenotype only for *ZmIAA10* which corresponds to *rootless with undetectable meristem 1* (*rum1*). The mutant does not initiate seminal roots and lacks lateral roots at the primary root, while primary and shoot-borne roots were not affected [25]. In the present study, *ZmIAA10* displays the highest expression in stele and cortex tissues of three-day old primary roots. This is consistent with the observed phenotype because lateral roots are initiated from pericycle cells of the stele and from endodermis cells of the cortex [45]. Moreover, *ZmIAA10*/*rum1* displays significantly lower expression in the elongation zone than in any root-type or tissue. 

When *Aux/IAA* gene expression was compared pairwise between the four major maize root-types primary, seminal, lateral, and crown roots, 55 differential gene expression patterns were observed among the 30 maize *Aux/IAA* genes. Remarkably, among these differential expression patterns there was a strong bias with respect to expression levels between the different roots types with the tendency: crown roots > seminal roots > primary roots > lateral roots. Since this is a very general trend this might reflect differential control of auxin signal transduction in the molecular context of different root-types. Root-type-specific expression levels of *Aux/IAA* genes are controlled by upstream factors that bind to AuxRE in the promoter of *Aux/IAA* genes. Abundance of *Aux/IAA* transcripts and their proteins also affects the activity of downstream genes and thus contributes to the specific forms and functions of the different root-types of maize.

In 27 of 30 maize *Aux/IAA* genes surveyed in the present study, preferential expression in cortex and stele tissues of the differentiation zone compared to the meristematic and elongation zones was observed. This expression pattern correlates with auxin response in maize roots as visualized by DR5:RFP [46]. DR5:RFP reports sites where strong Aux/IAA protein degradation occurs [46]. These sites typically correlate with auxin maxima which enhance the transcription of *Aux/IAA* genes. Hence, DR5:RFP is also an indirect sensor for *Aux/IAA* transcriptional activity. In maize roots DR5:RFP maxima were observed in metaxylem elements and phloem poles in the stele [46]. Moreover, at early stages of lateral root development auxin response maxima were detected in pericycle and endodermis cells [46]. Remarkably, DR5:RFP also displays a strong signal in the meristematic zone of maize roots [46]. In this zone, only moderate *Aux/IAA* transcription was observed for most members of the gene family. However, while the DR5:RFP peak was mainly localized in the root cap, expression was surveyed in root tips that went beyond the root cap and also included the meristematic zone.

### Auxin induced gene expression

Auxin-responsive (AuxRE) *cis*-elements are characteristic of the promoters of auxin-responsive genes [21]. Promoter analyses illustrated that all maize *Aux/IAA* genes contain canonical auxin-response elements or their core sequence. In contrast, in the promoter region of *Aux/IAA* genes of *Vitis vinifera* no AuxRE motifs were identified [47]. Similarly, in *Arabidopsis* only the promoters of *AtIAA26* and *AtIAA29* contain an AuxRE motif [48]. Consequently, auxin-induced gene expression may be directed by tissue-specific factors different than Aux/IAA proteins in these plants [47,48]. Other promoter elements like MYB and bZIP related binding sites play a role in auxin-mediated transcription [49,50]. Similar results were presented for a putative ocs element in *Arabidopsis* [51]. Consistent with the presence of AuxRE or their core elements, 27 of 28 expressed maize *Aux/IAA* genes were auxin inducible in qRT-PCR experiments. Similarly, in rice 24 of 29 expressed Aux/IAA genes were auxin inducible [44]. The kinetics of *Aux/IAA* gene expression is unique and depends on the variability of the regulation of free auxin, tissue-specific auxin receptors and different regulation of transcriptional and posttranscriptional events [14]. In maize, two auxin-dependent expression patterns were observed after αNAA treatment. While 22 *Aux/IAA* genes were constantly induced over time, five *Aux/IAA* genes showed a significant decrease after an initial increase in expression. Similarly, in rice 12 of 24 Aux/IAA genes displayed continuously increased expression during the time course while 12 genes displayed decreased expression at later time points [44]. In tomato, up-regulation of transcript levels of 17 of 19 *Sl-Aux/IAA* genes was detected in seedlings upon auxin treatment [12].

In summary, the detailed analysis of the maize *Aux/IAA* genes provides novel insights into the organization and expression of this large gene family that plays a crucial role in auxin signal transduction and thus the regulation of maize development. Moreover, tissue and root-type-specific expression profiles and induction studies provide interesting starting points for genetic analyses of candidate genes that might be involved in the initiation, emergence or specification of specific root-types in the complex maize root stock.

## Supporting Information

Figure S1 **Alignment of the maize Aux/IAA protein sequences.** Aux/IAA protein sequences were compared by multiple alignments of the four conserved domains with ClustalW. Differences in the amino acid sequences of domain III, which distinguish class A and class B Aux/IAA proteins (see Figure 1) are boxed. The four domains are highlighted.
(PDF)

Figure S2 **Phylogenetic reconstruction of the Aux/IAA protein families in different monocot species.** Phylogenetic reconstruction of maize (*Zea mays*, Zm) sorghum (*Sorghum bicolor*, Sb), and rice (*Oryza sativa*, Os) Aux/IAA protein families in an unrooted tree with the neighbor-joining algorithm of MEGA5. Monocot specific clades are encircled. The values associated to each branch are bootstrap percentages. The size bar indicates sequence divergence: 0.05 = 5%.
(PDF)

Figure S3 **Summary of *Aux/IAA* gene expression patterns in maize.** Gene expression patterns obtained by qRT-PCR experiments in root and shoot tissues. Expression values in whole roots are highlighted in black, expression in primary root tissues in dark grey, and expression in shoot organs in light grey. *ZmIAA10*, *ZmIAA13*, and *ZmIAA30* did not display any expression in shoot tissues. l: light, d:dark, N.D.: no expression detected.
(PDF)

Figure S4 **Summary of pairwise Student´s t-tests of *Aux/IAA* gene expression comparisons in root and shoot tissues.** Pairwise comparison of differential gene expression patterns between the various roots and shoot tissues by a two-sided Student´s t-test. Different significance levels are highlighted in color. Red: *p* ≤0.05; yellow: *p* ≤0.01; green: *p* ≤0.001. N. D. Expression was not detected in one of these tissues.
(PDF)

Figure S5 **Summary of maize *Aux/IAA* gene induction by αNAA.** Auxin induction patterns of the maize *Aux/IAA* genes determined by qRT-PCR in the differentiation zone of 5-day-old maize primary roots after 5 µM αNAA (**α**-Naphthalene Acetic Acid) treatment over three hours. A summary of these results in provided in Figure 4.
(PDF)

Table S1 **Oligonucleotide primers used in the present study.**
(XLSX)

Table S2 **Promoter analyses of 3 kb upstream of the ATG start codon of maize *Aux/IAA* genes.**
(XLSX)
