# Supplementary material for: The Maize (Zea mays L.) AUXIN/INDOLE-3-ACETIC ACID Gene Family: Phylogeny, Synteny, and Unique Root-Type and Tissue-Specific Expression Patterns during Development
Source: PLoS One. 2013 Nov 1;8(11):e78859. doi: 10.1371/journal.pone.0078859 (PMC3815225; doi:10.1371/journal.pone.0078859)
Supplement: Figure S1 — Alignment of the maize Aux/IAA protein sequences. Aux/IAA protein sequences were compared by multiple alignments of the four conserved domains with ClustalW. Differences in the amino acid sequences of domain III, which distinguish class A and class B Aux/IAA proteins (see Figure 1) are boxed. The four domains are highlighted. (PDF) [file pone.0078859.s001.pdf]

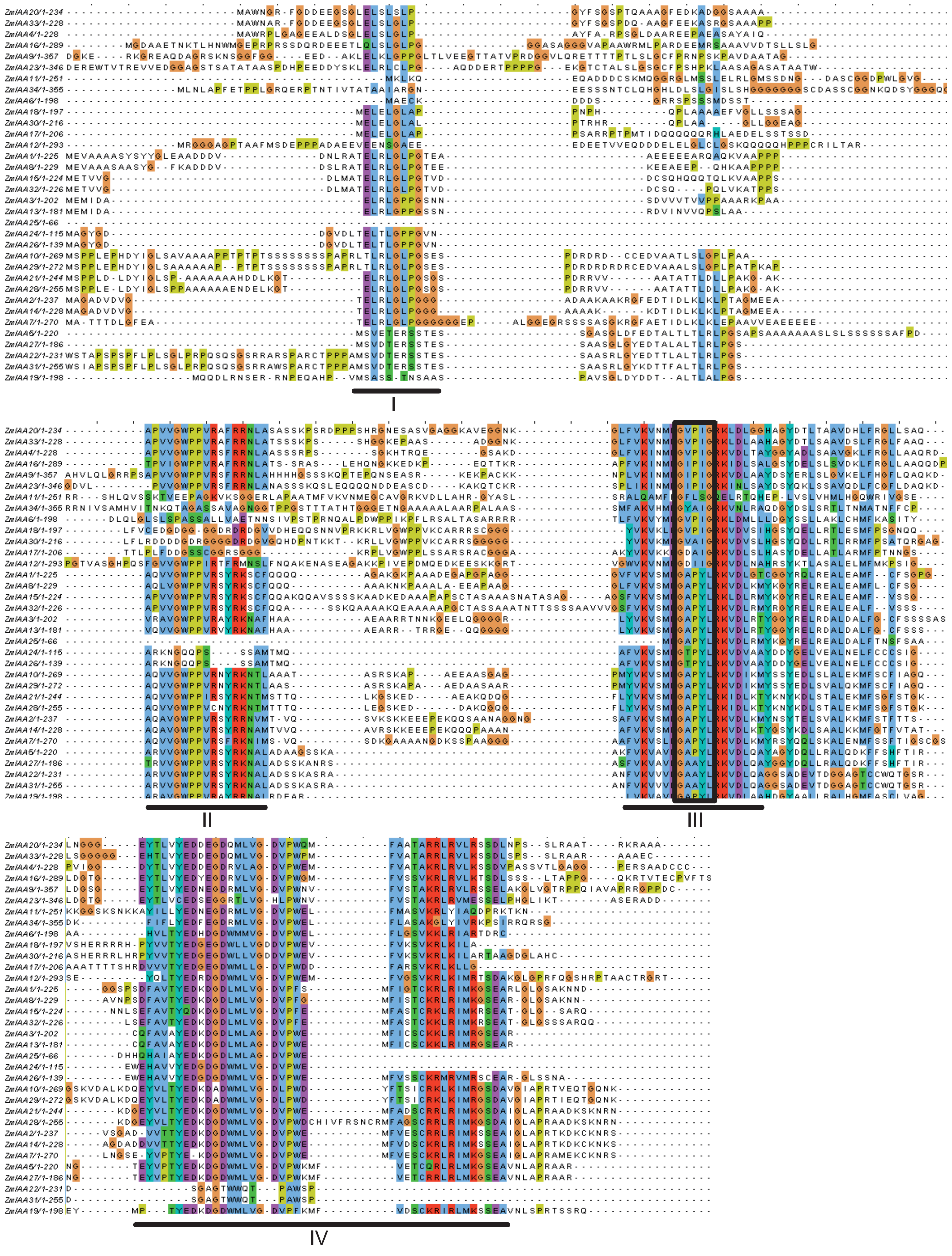

Figure S1
