# Supplementary figures and images for: The Maize (Zea mays L.) AUXIN/INDOLE-3-ACETIC ACID Gene Family: Phylogeny, Synteny, and Unique Root-Type and Tissue-Specific Expression Patterns during Development
Source: PLoS One. 2013 Nov 1;8(11):e78859. doi: 10.1371/journal.pone.0078859 (PMC3815225; doi:10.1371/journal.pone.0078859)

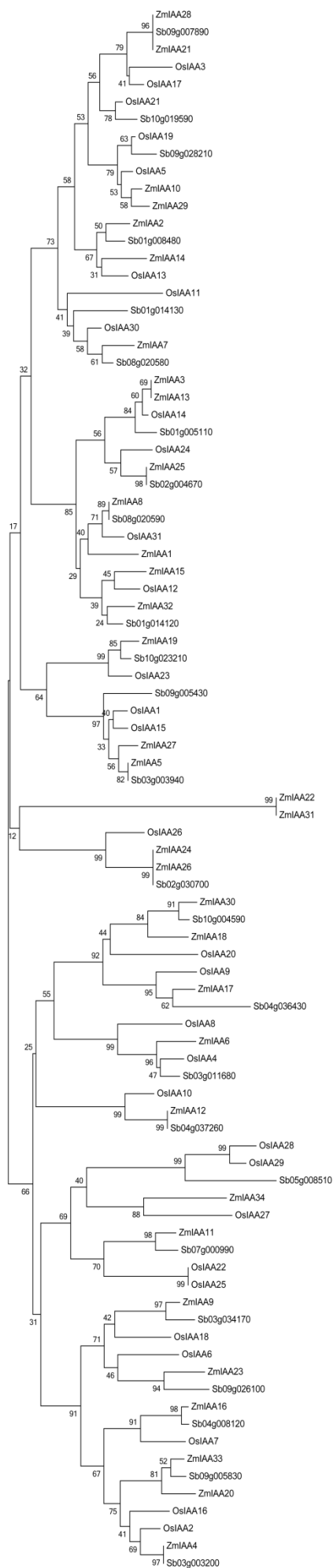

Figure S2

Supplement: Figure S2 — Phylogenetic reconstruction of the Aux/IAA protein families in different monocot species. Phylogenetic reconstruction of maize (Zea mays, Zm) sorghum (Sorghum bicolor, Sb), and rice (Oryza sativa, Os) Aux/IAA protein families in an unrooted tree with the neighbor-joining algorithm of MEGA5. Monocot specific clades are encircled. The values associated to each branch are bootstrap percentages. The size bar indicates sequence divergence: 0.05 = 5%. (PDF) [file pone.0078859.s002.pdf]

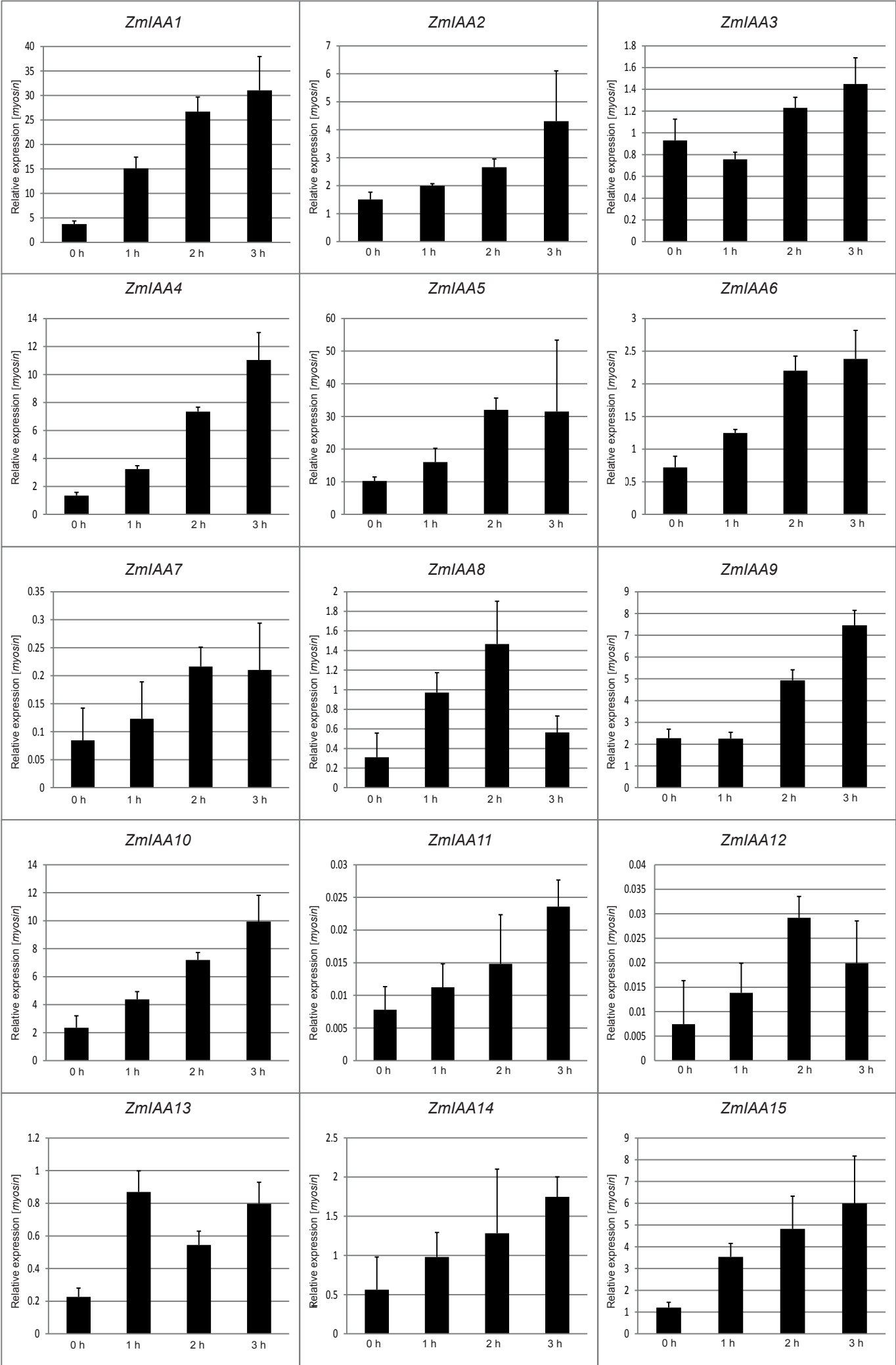

Figure S5-1

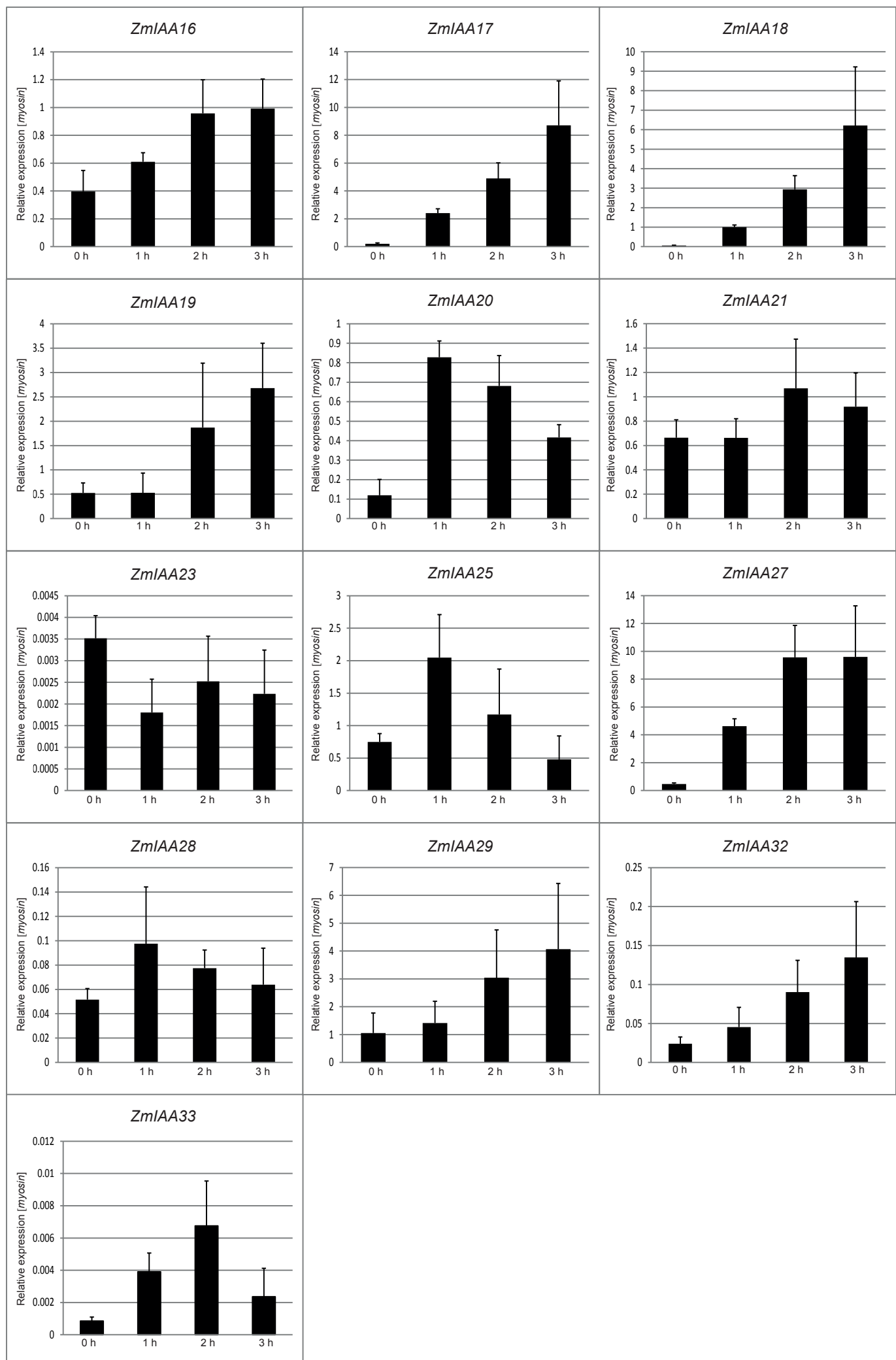

Figure S5-2

Supplement: Figure S5 — Summary of maize Aux/IAA gene induction by αNAA. Auxin induction patterns of the maize Aux/IAA genes determined by qRT-PCR in the differentiation zone of 5-day-old maize primary roots after 5 µM αNAA (α-Naphthalene Acetic Acid) treatment over three hours. A summary of these results in provided in Figure 4. (PDF) [file pone.0078859.s005.pdf]
