# Supplementary material for: The Maize (Zea mays L.) AUXIN/INDOLE-3-ACETIC ACID Gene Family: Phylogeny, Synteny, and Unique Root-Type and Tissue-Specific Expression Patterns during Development
Source: PLoS One. 2013 Nov 1;8(11):e78859. doi: 10.1371/journal.pone.0078859 (PMC3815225; doi:10.1371/journal.pone.0078859)
Supplement: Figure S3 — Summary of Aux/IAA gene expression patterns in maize. Gene expression patterns obtained by qRT-PCR experiments in root and shoot tissues. Expression values in whole roots are highlighted in black, expression in primary root tissues in dark grey, and expression in shoot organs in light grey. ZmIAA10, ZmIAA13, and ZmIAA30 did not display any expression in shoot tissues. l: light, d:dark, N.D.: no expression detected. (PDF) [file pone.0078859.s003.pdf]

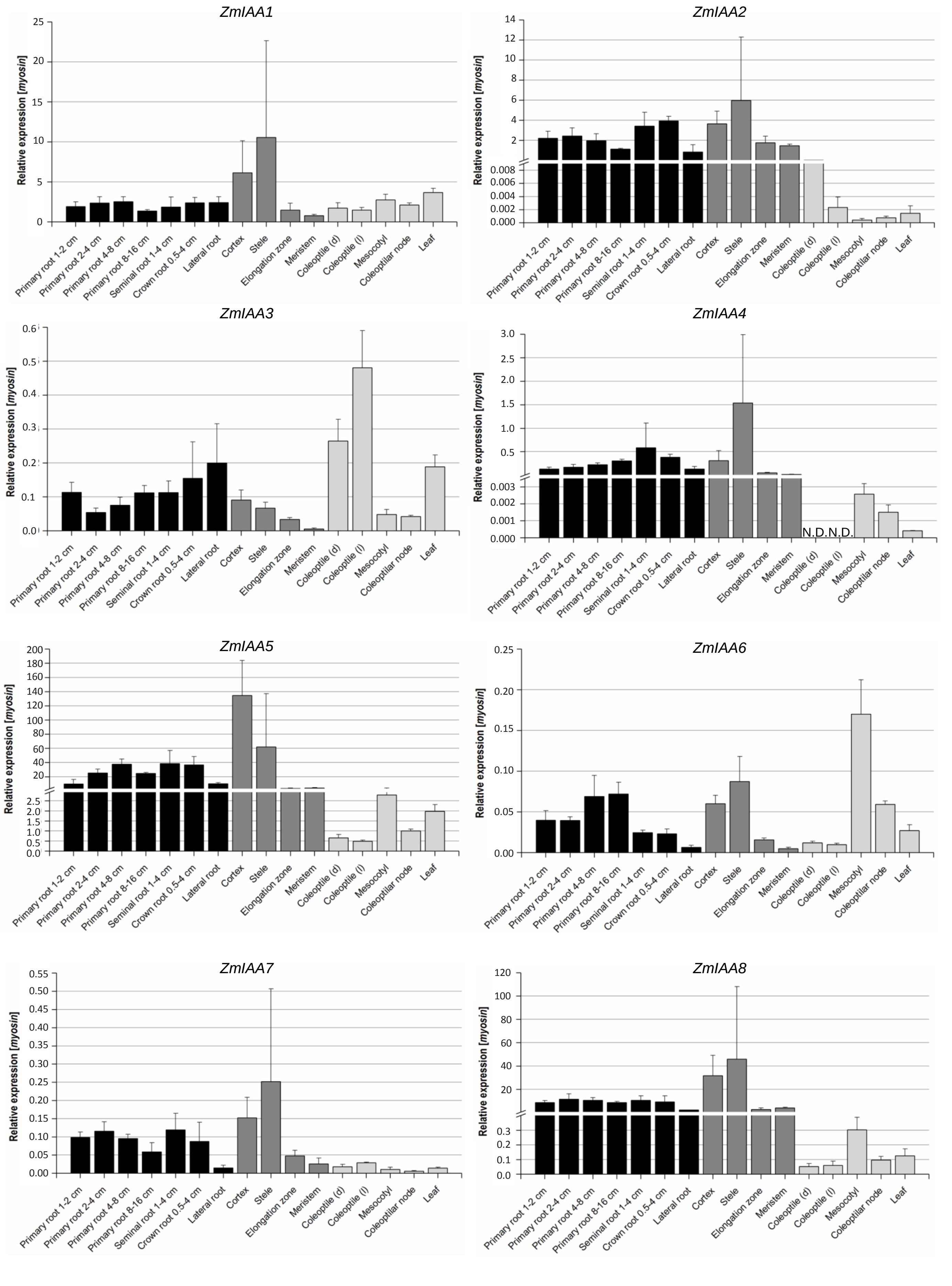
ZmIAA1
Relative expression [myosin]
25
20
15
10
5
0
Primary root 1-2 cm
Primary root 2-4 cm
Primary root 4-8 cm
Primary root 8-16 cm
Seminal root 1-4 cm
Crown root 0.5-4 cm
Lateral root
Cortex
Stele
Elongation zone
Meristem
Coleoptile (d)
Coleoptile (l)
Mesocotyl
Coleoptilar node
Leaf
ZmIAA2
Relative expression [myosin]
14
12
10
8
6
4
2
0.008
0.006
0.004
0.002
0.000
Primary root 1-2 cm
Primary root 2-4 cm
Primary root 4-8 cm
Primary root 8-16 cm
Seminal root 1-4 cm
Crown root 0.5-4 cm
Lateral root
Cortex
Stele
Elongation zone
Meristem
Coleoptile (d)
Coleoptile (l)
Mesocotyl
Coleoptilar node
Leaf
ZmIAA3
Relative expression [myosin]
0.6
0.5
0.4
0.3
0.2
0.1
0.0
Primary root 1-2 cm
Primary root 2-4 cm
Primary root 4-8 cm
Primary root 8-16 cm
Seminal root 1-4 cm
Crown root 0.5-4 cm
Lateral root
Cortex
Stele
Elongation zone
Meristem
Coleoptile (d)
Coleoptile (l)
Mesocotyl
Coleoptilar node
Leaf
ZmIAA4
Relative expression [myosin]
3.0
2.5
2.0
1.5
1.0
0.5
0.003
0.002
0.001
0.000
N.D.N.D.
Primary root 1-2 cm
Primary root 2-4 cm
Primary root 4-8 cm
Primary root 8-16 cm
Seminal root 1-4 cm
Crown root 0.5-4 cm
Lateral root
Cortex
Stele
Elongation zone
Meristem
Coleoptile (d)
Coleoptile (l)
Mesocotyl
Coleoptilar node
Leaf
ZmIAA5
Relative expression [myosin]
200
180
160
140
120
100
80
60
40
20
2.5
2.0
1.5
1.0
0.5
0.0
Primary root 1-2 cm
Primary root 2-4 cm
Primary root 4-8 cm
Primary root 8-16 cm
Seminal root 1-4 cm
Crown root 0.5-4 cm
Lateral root
Cortex
Stele
Elongation zone
Meristem
Coleoptile (d)
Coleoptile (l)
Mesocotyl
Coleoptilar node
Leaf
ZmIAA6
Relative expression [myosin]
0.25
0.20
0.15
0.10
0.05
0.00
Primary root 1-2 cm
Primary root 2-4 cm
Primary root 4-8 cm
Primary root 8-16 cm
Seminal root 1-4 cm
Crown root 0.5-4 cm
Lateral root
Cortex
Stele
Elongation zone
Meristem
Coleoptile (d)
Coleoptile (l)
Mesocotyl
Coleoptilar node
Leaf
ZmIAA7
Relative expression [myosin]
0.55
0.50
0.45
0.40
0.35
0.30
0.25
0.20
0.15
0.10
0.05
0.00
Primary root 1-2 cm
Primary root 2-4 cm
Primary root 4-8 cm
Primary root 8-16 cm
Seminal root 1-4 cm
Crown root 0.5-4 cm
Lateral root
Cortex
Stele
Elongation zone
Meristem
Coleoptile (d)
Coleoptile (l)
Mesocotyl
Coleoptilar node
Leaf
ZmIAA8
Relative expression [myosin]
120
100
80
60
40
20
0.3
0.2
0.1
0.0
Primary root 1-2 cm
Primary root 2-4 cm
Primary root 4-8 cm
Primary root 8-16 cm
Seminal root 1-4 cm
Crown root 0.5-4 cm
Lateral root
Cortex
Stele
Elongation zone
Meristem
Coleoptile (d)
Coleoptile (l)
Mesocotyl
Coleoptilar node
Leaf

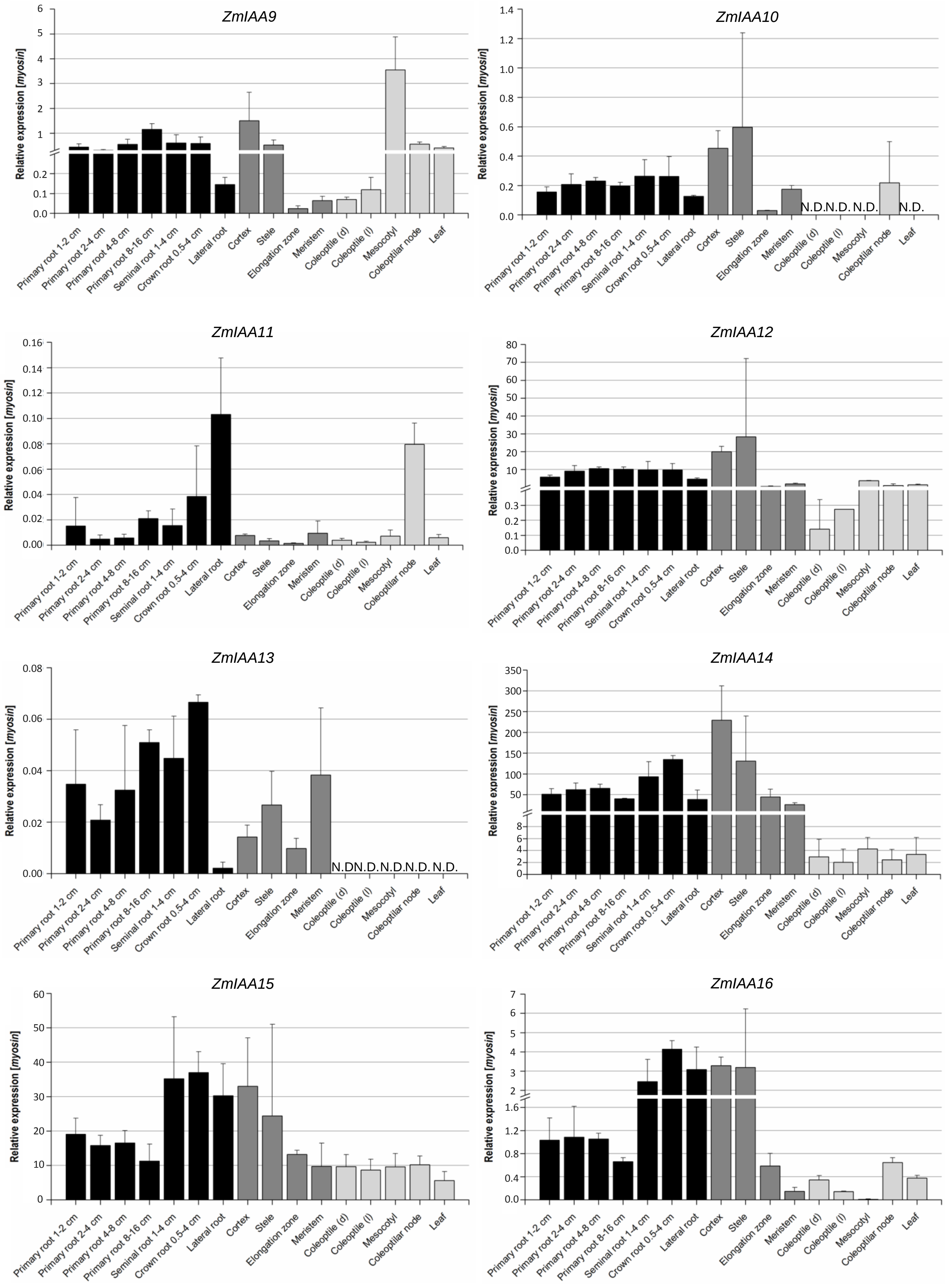
ZmIAA9
Relative expression [myosin]
Primary root 1-2 cm
Primary root 2-4 cm
Primary root 4-8 cm
Primary root 8-16 cm
Seminal root 1-4 cm
Crown root 0.5-4 cm
Lateral root
Cortex
Stele
Elongation zone
Meristem
Coleoptile (d)
Coleoptile (l)
Mesocotyl
Coleoptilar node
Leaf
ZmIAA10
N.D.N.D.N.D.
N.D.
ZmIAA11
ZmIAA12
ZmIAA13
N.DN.D.N.D.N.D. N.D.
ZmIAA14
ZmIAA15
ZmIAA16

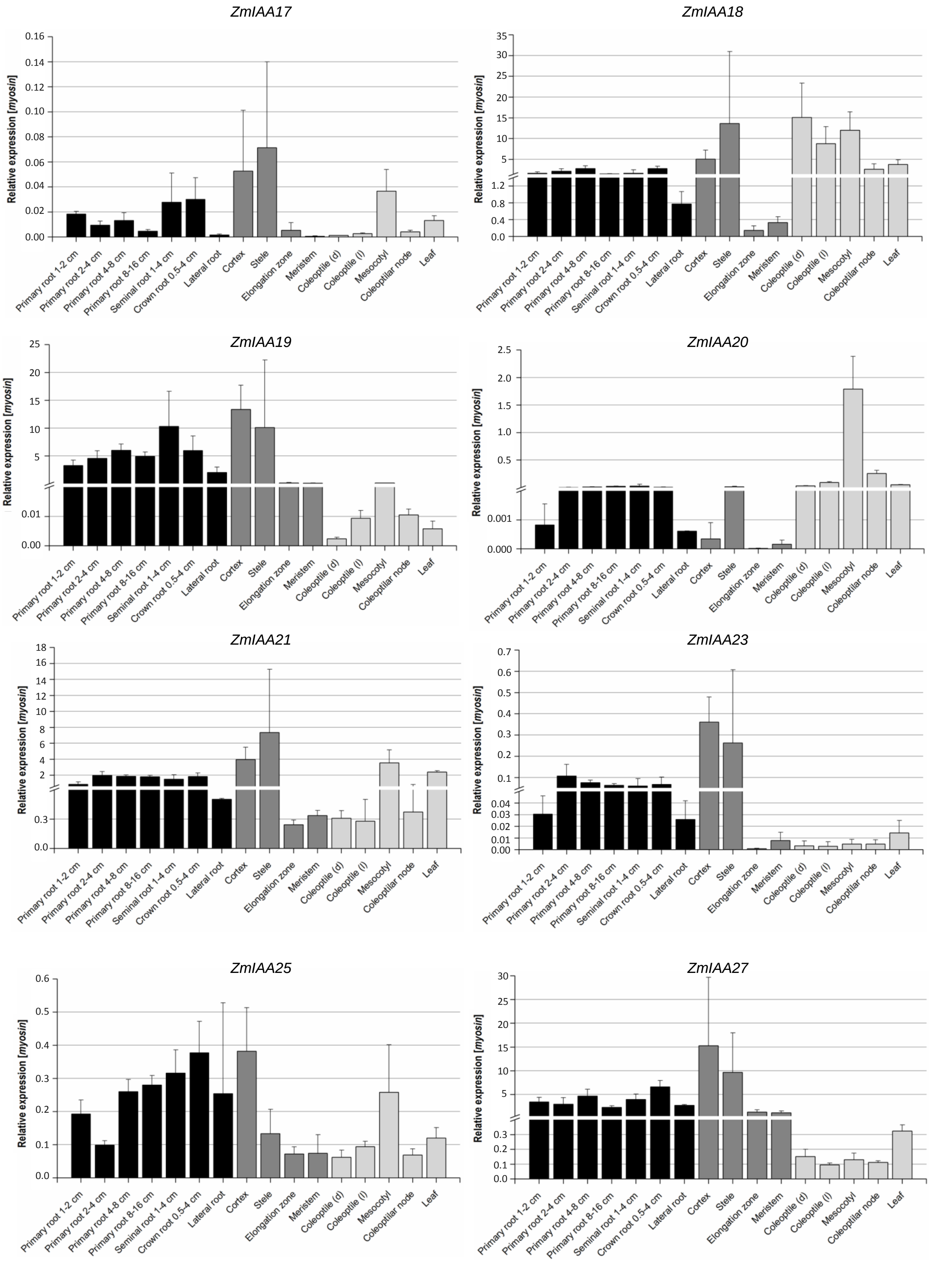
ZmIAA17
ZmIAA18
ZmIAA19
ZmIAA20
ZmIAA21
ZmIAA23
ZmIAA25
ZmIAA27
Relative expression [myosin]
Primary root 1-2 cm
Primary root 2-4 cm
Primary root 4-8 cm
Primary root 8-16 cm
Seminal root 1-4 cm
Crown root 0.5-4 cm
Lateral root
Cortex
Stele
Elongation zone
Meristem
Coleoptile (d)
Coleoptile (l)
Mesocotyl
Coleoptilar node
Leaf

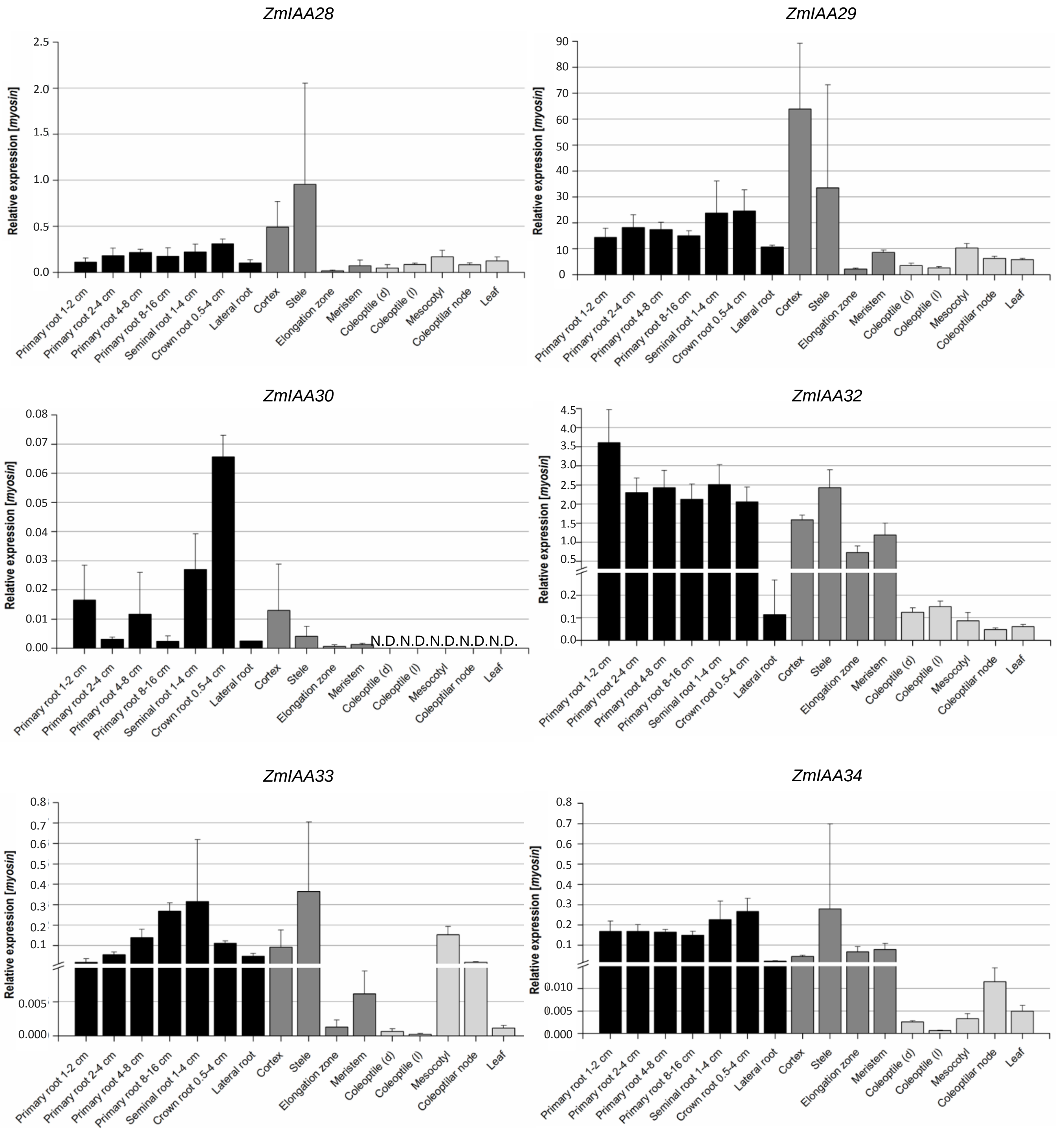

ZmIAA28
ZmIAA29
ZmIAA30
ZmIAA32
ZmIAA33
ZmIAA34
Relative expression [myosin]
N.D.N.D.N.D.N.D.N.D.N.D.
Primary root 1-2 cm
Primary root 2-4 cm
Primary root 4-8 cm
Primary root 8-16 cm
Seminal root 1-4 cm
Crown root 0.5-4 cm
Lateral root
Cortex
Stele
Elongation zone
Meristem
Coleoptile (d)
Coleoptile (l)
Mesocotyl
Coleoptilar node
Leaf

Figure S3
