# Supplementary material for: The Maize (Zea mays L.) AUXIN/INDOLE-3-ACETIC ACID Gene Family: Phylogeny, Synteny, and Unique Root-Type and Tissue-Specific Expression Patterns during Development
Source: PLoS One. 2013 Nov 1;8(11):e78859. doi: 10.1371/journal.pone.0078859 (PMC3815225; doi:10.1371/journal.pone.0078859)
Supplement: Figure S4 — Summary of pairwise Student´s t-tests of Aux/IAA gene expression comparisons in root and shoot tissues. Pairwise comparison of differential gene expression patterns between the various roots and shoot tissues by a two-sided Student´s t-test. Different significance levels are highlighted in color. Red: p ≤0.05; yellow: p ≤0.01; green: p ≤0.001. N. D. Expression was not detected in one of these tissues. (PDF) [file pone.0078859.s004.pdf]

ZmIAA1

Primary root 1-2 cm	Primary root 2-4 cm	Primary root 4-8 cm	Primary root 8-16 cm	Seminal root 1-4 cm	Crown root 0.5-4 cm	Stele	Cortex	Elongation zone	Meristem	Lateral root	Coleoptile (d)	Coleoptile (l)	Mesocotyl	Coleoptilar node	Leaf	
x	0.404	0.35	0.195	0.911	0.211	0.258	0.138	0.264	0.049	0.361	0.312	0.131	0.208	0.518	0.013	**Primary root 1-2 cm**
	x	0.638	0.103	0.537	0.936	0.275	0.113	0.254	0.03	0.894	0.263	0.164	0.592	0.625	0.122	**Primary root 2-4 cm**
		x	0.033	0.45	0.826	0.274	0.13	0.222	0.008	0.655	0.246	0.099	0.709	0.343	0.129	**Primary root 4-8 cm**
			x	0.479	0.034	0.229	0.094	0.864	0.008	0.323	0.349	0.521	0.042	0.002	0.005	**Primary root 8-16 cm**
				x	0.179	0.282	0.118	0.681	0.227	0.501	0.786	0.529	0.418	0.648	0.092	**Seminal root 1-4 cm**
					x	0.289	0.158	0.213	0.028	0.685	0.044	0.022	0.633	0.269	0.066	**Crown root 0.5-4 cm**
						x	0.576	0.223	0.199	0.392	0.257	0.236	0.265	0.263	0.33	**Stele**
							x	0.139	0.075	0.477	0.117	0.109	0.227	0.137	0.353	**Cortex**
								x	0.208	0.085	0.613	0.974	0.046	0.27	0.004	**Elongation zone**
									x	0.168	0.098	0.054	0.006	0.006	0.002	**Meristem**
										x	0.411	0.259	0.301	0.593	0.044	**Lateral root**
											x	0.335	0.202	0.242	0.02	**Coleoptile (d)**
												x	0.062	0.005	0.003	**Coleoptile (l)**
													x	0.25	0.033	**Mesocotyl**
														x	0.014	**Coleopilar node**
															x	**Leaf**

(pink)	*p* ≤0.05
(orange)	*p* ≤0.01
(green)	*p* ≤0.001
N.D.	not detectable

ZmIAA2

Primary root 1-2 cm	Primary root 2-4 cm	Primary root 4-8 cm	Primary root 8-16 cm	Seminal root 1-4 cm	Crown root 0.5-4 cm	Stele	Cortex	Elongation zone	Meristem	Lateral root	Coleoptile (d)	Coleoptile (l)	Mesocotyl	Coleoptilar node	Leaf	
x	0.776	0.674	0.058	0.146	0.053	0.338	0.19	0.097	0.117	0.47	0.008	0.008	0.008	0.008	0.008	**Primary root 1-2 cm**
	x	0.563	0.041	0.375	0.025	0.356	0.046	0.394	0.085	0.086	0.01	0.01	0.01	0.01	0.01	**Primary root 2-4 cm**
		x	0.116	0.124	0.016	0.267	0.159	0.676	0.294	0.486	0.011	0.011	0.011	0.011	0.011	**Primary root 4-8 cm**
			x	0.044	1E-03	0.226	0.024	0.165	0.294	0.606	1E-04	1E-04	1E-04	1E-04	1E-04	**Primary root 8-16 cm**
				x	0.525	0.531	0.831	0.115	0.078	0.141	0.015	0.015	0.015	0.015	0.015	**Seminal root 1-4 cm**
					x	0.566	0.615	0.029	0.003	0.042	4E-04	4E-04	4E-04	4E-04	4E-04	**Crown root 0.5-4 cm**
						x	0.561	0.271	0.247	0.385	0.156	0.156	0.156	0.156	0.156	**Stele**
							x	0.13	0.041	0.205	0.01	0.01	0.01	0.01	0.01	**Cortex**
								x	0.385	0.65	0.013	0.012	0.012	0.012	0.012	**Elongation zone**
									x	0.421	5E-04	4E-04	4E-04	4E-04	4E-04	**Meristem**
										x	0.354	0.349	0.349	0.349	0.349	**Lateral root**
											x	0.091	0.035	0.038	0.036	**Coleoptile (d)**
												x	0.11	0.167	0.52	**Coleoptile (l)**
													x	0.004	0.114	**Mesocotyl**
														x	0.214	**Coleopilar node**
															x	**Leaf**

ZmIAA3

Primary root 1-2 cm	Primary root 2-4 cm	Primary root 4-8 cm	Primary root 8-16 cm	Seminal root 1-4 cm	Crown root 0.5-4 cm	Stele	Cortex	Elongation zone	Meristem	Lateral root	Coleoptile (d)	Coleoptile (l)	Mesocotyl	Coleoptilar node	Leaf	
x	0.034	0.153	0.964	0.986	0.464	0.042	0.25	0.01	0.006	0.481	0.039	0.008	0.009	0.021	0.08	**Primary root 1-2 cm**
	x	0.297	0.018	0.082	0.126	0.42	0.051	0.016	0.006	0.301	0.011	0.004	0.583	0.204	0.008	**Primary root 2-4 cm**
		x	0.164	0.075	0.293	0.302	0.587	0.054	0.007	0.476	0.006	0.008	0.205	0.05	0.014	**Primary root 4-8 cm**
			x	0.978	0.494	0.08	0.222	0.006	0.007	0.371	0.015	0.004	0.01	0.007	0.006	**Primary root 8-16 cm**
				x	0.589	0.065	0.51	0.027	0.007	0.568	0.002	0.008	0.049	0.019	0.008	**Seminal root 1-4 cm**
					x	0.23	0.233	0.099	0.071	0.298	0.283	0.015	0.134	0.134	0.645	**Crown root 0.5-4 cm**
						x	0.331	0.042	0.004	0.417	0.008	0.007	0.187	0.049	0.011	**Stele**
							x	0.02	0.013	0.292	0.028	0.003	0.029	0.056	0.027	**Cortex**
								x	0.004	0.286	0.007	0.004	0.125	0.153	0.004	**Elongation zone**
									x	0.262	0.004	0.003	0.015	8E-05	0.002	**Meristem**
										x	0.361	0.088	0.294	0.31	0.983	**Lateral root**
											x	0.044	0.009	0.005	0.036	**Coleoptile (d)**
												x	0.003	0.004	0.008	**Coleoptile (l)**
													x	0.495	0.005	**Mesocotyl**
														x	0.003	**Coleopilar node**
															x	**Leaf**

ZmIAA4

Primary root 1-2 cm	Primary root 2-4 cm	Primary root 4-8 cm	Primary root 8-16 cm	Seminal root 1-4 cm	Crown root 0.5-4 cm	Stele	Cortex	Elongation zone	Meristem	Lateral root	Coleoptile (d)	Coleoptile (l)	Mesocotyl	Coleoptilar node	Leaf	
x	0.443	0.032	0.016	0.162	0.013	0.152	0.222	0.025	0.013	0.318	N.D.	0.5	0.007	0.007	0.126	**Primary root 1-2 cm**
	x	0.237	0.002	0.228	0.003	0.157	0.202	0.021	0.009	0.57	N.D.	0.5	0.008	0.008	0.155	**Primary root 2-4 cm**
		x	0.078	0.253	0.045	0.175	0.431	8E-04	0.002	0.469	N.D.	0.5	0.001	0.001	0.075	**Primary root 4-8 cm**
			x	0.364	0.021	0.187	0.942	0.001	0.002	0.205	N.D.	0.5	5E-04	5E-04	0.053	**Primary root 8-16 cm**
				x	0.498	0.373	0.405	0.132	0.12	0.339	N.D.	0.5	0.112	0.112	0.09	**Seminal root 1-4 cm**
					x	0.204	0.54	0.003	0.001	0.129	N.D.	0.5	0.002	0.001	0.035	**Crown root 0.5-4 cm**
						x	0.22	0.135	0.126	0.283	N.D.	0.5	0.125	0.125	0.277	**Stele**
							x	0.082	0.068	0.637	N.D.	0.5	0.061	0.06	0.43	**Cortex**
								x	0.026	0.294	N.D.	0.5	0.004	0.004	0.13	**Elongation zone**
									x	0.185	N.D.	0.5	0.028	0.021	0.088	**Meristem**
										x	N.D.	0.5	0.173	0.173	0.172	**Lateral root**
											x	N.D.	N.D.	N.D.	N.D.	**Coleoptile (d)**
												x	0.5	0.5	0.5	**Coleoptile (l)**
													x	0.078	0.09	**Mesocotyl**
														x	0.083	**Coleopilar node**
															x	**Leaf**

	p ≤0.05
	p ≤0.01
	p ≤0.001
N.D.	not detectable

ZmIAA5

Primary root 1-2 cm	Primary root 2-4 cm	Primary root 4-8 cm	Primary root 8-16 cm	Seminal root 1-4 cm	Crown root 0.5-4 cm	Stele	Cortex	Elongation zone	Meristem	Lateral root	Coleoptile (d)	Coleoptile (l)	Mesocotyl	Coleoptilar node	Leaf	
x	0.018	0.019	0.024	0.091	0.009	0.249	0.016	0.142	0.152	0.316	0.07	0.062	0.109	0.072	0.092	**Primary root 1-2 cm**
	x	0.039	0.893	0.232	0.071	0.422	0.017	0.004	0.004	0.272	0.003	0.003	0.005	0.003	0.004	**Primary root 2-4 cm**
		x	0.041	0.894	0.879	0.591	0.024	0.002	0.003	0.118	0.002	0.002	0.003	0.002	0.002	**Primary root 4-8 cm**
			x	0.234	0.17	0.391	0.022	1E-04	0.003	0.015	3E-05	4E-05	4E-05	4E-05	3E-05	**Primary root 8-16 cm**
				x	0.879	0.635	0.023	0.031	0.035	0.276	0.026	0.026	0.034	0.027	0.029	**Seminal root 1-4 cm**
					x	0.575	0.019	0.012	0.011	0.247	0.01	0.009	0.011	0.01	0.011	**Crown root 0.5-4 cm**
						x	0.323	0.217	0.221	0.468	0.202	0.2	0.21	0.203	0.207	**Stele**
							x	0.013	0.013	0.306	0.012	0.012	0.013	0.012	0.013	**Cortex**
								x	0.129	0.096	0.003	0.001	0.434	0.003	0.019	**Elongation zone**
									x	0.149	0.003	0.001	0.107	0.002	0.007	**Meristem**
										x	0.067	0.066	0.037	0.064	0.053	**Lateral root**
											x	0.193	0.034	0.039	0.007	**Coleoptile (d)**
												x	0.026	8E-04	0.002	**Coleoptile (l)**
													x	0.043	0.161	**Mesocotyl**
														x	0.004	**Coleopilar node**
															x	**Leaf**

ZmIAA6

Primary root 1-2 cm	Primary root 2-4 cm	Primary root 4-8 cm	Primary root 8-16 cm	Seminal root 1-4 cm	Crown root 0.5-4 cm	Stele	Cortex	Elongation zone	Meristem	Lateral root	Coleoptile (d)	Coleoptile (l)	Mesocotyl	Coleoptilar node	Leaf	
x	0.938	0.204	0.003	0.101	0.141	0.086	0.274	0.026	0.008	0.197	0.016	0.017	0.01	0.082	0.178	**Primary root 1-2 cm**
	x	0.126	0.024	0.017	0.012	0.042	0.069	0.001	3E-04	0.054	4E-04	0.002	0.011	0.004	0.064	**Primary root 2-4 cm**
		x	0.886	0.032	0.026	0.442	0.937	0.025	0.018	0.275	0.025	0.017	0.011	0.459	0.058	**Primary root 4-8 cm**
			x	0.008	0.014	0.52	0.442	0.004	0.018	0.081	0.004	0.004	0.021	0.234	0.021	**Primary root 8-16 cm**
				x	0.664	0.03	0.012	0.005	0.003	0.179	0.015	0.002	0.006	2E-04	0.624	**Seminal root 1-4 cm**
					x	0.02	0.006	0.065	0.01	0.286	0.037	0.02	0.007	4E-05	0.491	**Crown root 0.5-4 cm**
						x	0.186	0.02	0.012	0.07	0.014	0.015	0.091	0.153	0.02	**Stele**
							x	0.011	0.013	0.138	0.017	0.012	0.009	0.858	0.075	**Cortex**
								x	0.004	0.241	0.104	0.04	0.006	1E-04	0.09	**Elongation zone**
									x	0.64	4E-04	0.051	0.005	2E-04	0.008	**Meristem**
										x	0.101	0.39	0.01	0.081	0.029	**Lateral root**
											x	0.301	0.006	3E-04	0.017	**Coleoptile (d)**
												x	0.004	2E-04	0.015	**Coleoptile (l)**
													x	0.015	0.007	**Mesocotyl**
														x	0.006	**Coleopilar node**
															x	**Leaf**

ZmIAA7

Primary root 1-2 cm	Primary root 2-4 cm	Primary root 4-8 cm	Primary root 8-16 cm	Seminal root 1-4 cm	Crown root 0.5-4 cm	Stele	Cortex	Elongation zone	Meristem	Lateral root	Coleoptile (d)	Coleoptile (l)	Mesocotyl	Coleoptilar node	Leaf	
x	0.417	0.127	0.034	0.333	0.708	0.325	0.129	0.019	0.007	0.058	0.003	0.004	0.003	0.001	0.002	**Primary root 1-2 cm**
	x	0.326	0.043	0.921	0.304	0.359	0.252	0.005	0.008	0.108	0.003	0.006	0.002	0.003	0.004	**Primary root 2-4 cm**
		x	0.044	0.294	0.78	0.317	0.116	0.019	0.006	0.062	0.002	0.002	0.002	6E-04	0.001	**Primary root 4-8 cm**
			x	0.114	0.389	0.218	0.039	0.28	0.006	0.047	0.036	0.104	0.032	0.021	0.033	**Primary root 8-16 cm**
				x	0.463	0.412	0.434	0.089	0.039	0.029	0.029	0.031	0.023	0.016	0.02	**Seminal root 1-4 cm**
					x	0.349	0.007	0.215	0.145	0.473	0.076	0.116	0.063	0.055	0.072	**Crown root 0.5-4 cm**
						x	0.551	0.199	0.158	0.363	0.159	0.176	0.151	0.147	0.156	**Stele**
							x	0.029	0.029	0.29	0.017	0.023	0.016	0.014	0.017	**Cortex**
								x	0.046	0.116	0.008	0.087	0.009	0.01	0.016	**Elongation zone**
									x	0.222	0.289	0.741	0.118	0.064	0.17	**Meristem**
										x	0.352	0.158	0.892	0.277	0.836	**Lateral root**
											x	0.031	0.016	0.022	0.211	**Coleoptile (d)**
												x	0.003	3E-04	0.001	**Coleoptile (l)**
													x	0.131	0.251	**Mesocotyl**
														x	5E-04	**Coleopilar node**
															x	**Leaf**

(pink)	*p* ≤0.05
(orange)	*p* ≤0.01
(green)	*p* ≤0.001
N.D.	not detectable

ZmIAA8

Primary root 1-2 cm	Primary root 2-4 cm	Primary root 4-8 cm	Primary root 8-16 cm	Seminal root 1-4 cm	Crown root 0.5-4 cm	Stele	Cortex	Elongation zone	Meristem	Lateral root	Coleoptile (d)	Coleoptile (l)	Mesocotyl	Coleoptilar node	Leaf	
x	0.354	0.319	0.988	0.439	0.874	0.309	0.079	0.022	0.007	0.146	0.002	0.002	0.002	0.002	0.002	**Primary root 1-2 cm**
	x	0.786	0.228	0.812	0.014	0.357	0.061	0.029	0.052	0.203	0.015	0.015	0.016	0.015	0.015	**Primary root 2-4 cm**
		x	0.238	0.973	0.708	0.348	0.116	0.005	0.013	0.022	0.003	0.003	0.003	0.003	0.003	**Primary root 4-8 cm**
			x	0.436	0.839	0.32	0.074	0.003	0.013	0.075	3E-04	3E-04	4E-04	3E-04	3E-04	**Primary root 8-16 cm**
				x	0.758	0.354	0.117	0.016	0.049	0.002	0.012	0.012	0.013	0.012	0.012	**Seminal root 1-4 cm**
					x	0.328	0.045	0.093	0.159	0.251	0.041	0.042	0.044	0.042	0.042	**Crown root 0.5-4 cm**
						x	0.701	0.267	0.268	0.429	0.238	0.238	0.24	0.238	0.238	**Stele**
							x	0.043	0.053	0.222	0.036	0.036	0.037	0.036	0.036	**Cortex**
								x	0.253	0.94	0.026	0.028	0.035	0.029	0.031	**Elongation zone**
									x	0.213	1E-03	9E-04	0.001	8E-04	8E-04	**Meristem**
										x	0.01	0.018	0.021	0.022	0.029	**Lateral root**
											x	0.6	0.017	0.11	0.086	**Coleoptile (d)**
												x	0.022	0.086	0.038	**Coleoptile (l)**
													x	0.018	0.042	**Mesocotyl**
														x	0.103	**Coleopilar node**
															x	**Leaf**

ZmIAA9

Primary root 1-2 cm	Primary root 2-4 cm	Primary root 4-8 cm	Primary root 8-16 cm	Seminal root 1-4 cm	Crown root 0.5-4 cm	Stele	Cortex	Elongation zone	Meristem	Lateral root	Coleoptile (d)	Coleoptile (l)	Mesocotyl	Coleoptilar node	Leaf	
x	0.137	0.472	0.02	0.437	0.447	0.204	0.141	0.006	0.007	0.17	0.01	0.005	0.015	0.298	0.711	**Primary root 1-2 cm**
	x	0.128	0.005	0.181	0.139	0.131	0.128	1E-04	5E-04	0.018	6E-04	0.005	0.016	0.013	0.081	**Primary root 2-4 cm**
		x	0.041	0.746	0.796	0.859	0.23	0.014	0.017	0.33	0.016	0.033	0.028	0.895	0.264	**Primary root 4-8 cm**
			x	0.024	0.077	0.035	0.574	0.002	0.017	0.132	0.003	0.005	0.041	0.018	0.004	**Primary root 8-16 cm**
				x	0.929	0.647	0.189	0.038	0.044	0.082	0.047	0.078	0.031	0.809	0.293	**Seminal root 1-4 cm**
					x	0.781	0.279	0.024	0.031	0.201	0.027	0.037	0.025	0.842	0.251	**Crown root 0.5-4 cm**
						x	0.145	0.013	0.015	0.237	0.02	0.02	0.017	0.781	0.422	**Stele**
							x	0.08	0.084	0.472	0.088	0.092	0.043	0.222	0.158	**Cortex**
								x	0.004	0.146	0.006	0.034	0.013	0.001	0.002	**Elongation zone**
									x	0.22	0.602	0.123	0.013	0.002	0.004	**Meristem**
										x	0.296	0.738	0.087	0.079	0.042	**Lateral root**
											x	0.182	0.013	9E-04	0.002	**Coleoptile (d)**
												x	0.012	0.004	0.016	**Coleoptile (l)**
													x	0.022	0.019	**Mesocotyl**
														x	0.018	**Coleopilar node**
															x	**Leaf**

ZmIAA10

Primary root 1-2 cm	Primary root 2-4 cm	Primary root 4-8 cm	Primary root 8-16 cm	Seminal root 1-4 cm	Crown root 0.5-4 cm	Stele	Cortex	Elongation zone	Meristem	Lateral root	Coleoptile (d)	Coleoptile (l)	Mesocotyl	Coleoptilar node	Leaf	
x	0.357	0.001	0.231	0.074	0.272	0.277	0.01	0.005	0.056	0.752	N.D.	N.D.	N.D.	0.718	N.D.	**Primary root 1-2 cm**
	x	0.645	0.67	0.536	0.203	0.329	0.03	0.015	0.564	0.337	N.D.	N.D.	N.D.	0.979	N.D.	**Primary root 2-4 cm**
		x	0.235	0.513	0.703	0.344	0.027	5E-04	0.002	0.076	N.D.	N.D.	N.D.	0.97	N.D.	**Primary root 4-8 cm**
			x	0.376	0.32	0.305	0.024	8E-04	0.002	0.132	N.D.	N.D.	N.D.	0.91	N.D.	**Primary root 8-16 cm**
				x	0.991	0.423	0.023	0.026	0.381	0.008	N.D.	N.D.	N.D.	0.949	N.D.	**Seminal root 1-4 cm**
					x	0.401	0.11	0.041	0.347	0.336	N.D.	N.D.	N.D.	0.461	N.D.	**Crown root 0.5-4 cm**
						x	0.732	0.006	0.339	0.42	N.D.	N.D.	N.D.	0.459	N.D.	**Stele**
							x	0.006	0.071	0.244	N.D.	N.D.	N.D.	0.257	N.D.	**Cortex**
								x	0.011	0.034	N.D.	N.D.	N.D.	0.365	N.D.	**Elongation zone**
									x	0.115	N.D.	N.D.	N.D.	0.818	N.D.	**Meristem**
										x	N.D.	N.D.	N.D.	0.684	N.D.	**Lateral root**
											x	N.D.	N.D.	N.D.	N.D.	**Coleoptile (d)**
												x	N.D.	N.D.	N.D.	**Coleoptile (l)**
													x	N.D.	N.D.	**Mesocotyl**
														x	N.D.	**Coleopilar node**
															x	**Leaf**

(pink)	$p \leq 0.05$
(orange)	$p \leq 0.01$
(green)	$p \leq 0.001$
N.D.	not detectable

ZmIAA11

Primary root 1-2 cm	Primary root 2-4 cm	Primary root 4-8 cm	Primary root 8-16 cm	Seminal root 1-4 cm	Crown root 0.5-4 cm	Stele	Cortex	Elongation zone	Meristem	Lateral root	Coleoptile (d)	Coleoptile (l)	Mesocotyl	Coleoptilar node	Leaf	
x	0.439	0.457	0.636	0.986	0.096	0.352	0.523	0.326	0.695	0.189	0.377	0.322	0.503	0.001	0.429	**Primary root 1-2 cm**
	x	0.949	0.008	0.406	0.241	0.662	0.534	0.249	0.493	0.215	0.623	0.329	0.882	0.03	0.595	**Primary root 2-4 cm**
		x	0.04	0.265	0.196	0.331	0.043	0.071	0.572	0.198	0.094	0.07	0.314	0.003	0.996	**Primary root 4-8 cm**
			x	0.484	0.43	0.007	0.038	0.002	0.572	0.241	0.018	0.009	0.079	0.009	0.008	**Primary root 8-16 cm**
				x	0.418	0.313	0.538	0.266	0.097	0.136	0.198	0.148	0.364	0.013	0.22	**Seminal root 1-4 cm**
					x	0.216	0.373	0.208	0.288	0.31	0.175	0.161	0.202	0.064	0.187	**Crown root 0.5-4 cm**
						x	0.24	0.134	0.352	0.186	0.854	0.44	0.387	0.02	0.008	**Stele**
							x	0.103	0.618	0.207	0.024	0.035	0.033	0.058	0.648	**Cortex**
								x	0.274	0.189	0.107	0.178	0.212	0.022	0.032	**Elongation zone**
									x	0.156	0.388	0.256	0.753	0.008	0.463	**Meristem**
										x	0.197	0.195	0.197	0.412	0.189	**Lateral root**
											x	0.052	0.164	0.002	0.35	**Coleoptile (d)**
												x	0.121	0.003	0.073	**Coleoptile (l)**
													x	0.002	0.689	**Mesocotyl**
														x	0.003	**Coleopilar node**
															x	**Leaf**

ZmIAA12

Primary root 1-2 cm	Primary root 2-4 cm	Primary root 4-8 cm	Primary root 8-16 cm	Seminal root 1-4 cm	Crown root 0.5-4 cm	Stele	Cortex	Elongation zone	Meristem	Lateral root	Coleoptile (d)	Coleoptile (l)	Mesocotyl	Coleoptilar node	Leaf	
x	0.062	0.003	0.004	0.093	0.072	0.251	4E-04	0.007	0.031	0.205	0.014	N.D.	0.14	0.157	0.123	**Primary root 1-2 cm**
	x	0.687	0.181	0.836	0.716	0.335	0.003	0.01	0.018	0.374	0.187	N.D.	0.299	0.068	0.239	**Primary root 2-4 cm**
		x	0.256	0.564	0.895	0.327	0.004	2E-04	3E-04	0.023	0.052	N.D.	0.061	0.002	0.032	**Primary root 4-8 cm**
			x	0.365	0.256	0.357	0.004	4E-04	3E-04	0.116	0.04	N.D.	0.074	0.006	0.068	**Primary root 8-16 cm**
				x	0.772	0.333	0.002	0.04	0.065	0.901	0.089	N.D.	0.537	0.191	0.203	**Seminal root 1-4 cm**
					x	0.34	0.007	0.015	0.022	0.335	0.181	N.D.	0.274	0.052	0.226	**Crown root 0.5-4 cm**
						x	0.649	0.21	0.215	0.49	0.465	N.D.	0.487	0.296	0.471	**Stele**
							x	5E-04	9E-04	0.112	0.072	N.D.	0.093	0.012	0.088	**Cortex**
								x	0.038	0.082	0.103	N.D.	0.032	0.272	0.182	**Elongation zone**
									x	0.01	0.225	N.D.	0.1	0.389	0.646	**Meristem**
										x	0.083	N.D.	0.249	0.009	0.046	**Lateral root**
											x	N.D.	0.038	0.231	0.166	**Coleoptile (d)**
												x	N.D.	N.D.	N.D.	**Coleoptile (l)**
													x	0.1	0.043	**Mesocotyl**
														x	0.737	**Coleopilar node**
															x	**Leaf**

ZmIAA13

Primary root 1-2 cm	Primary root 2-4 cm	Primary root 4-8 cm	Primary root 8-16 cm	Seminal root 1-4 cm	Crown root 0.5-4 cm	Stele	Cortex	Elongation zone	Meristem	Lateral root	Coleoptile (d)	Coleoptile (l)	Mesocotyl	Coleoptilar node	Leaf	
x	0.301	0.914	0.148	0.527	0.18	0.552	0.281	0.255	0.899	0.252	N.D.	N.D.	N.D.	N.D.	N.D.	**Primary root 1-2 cm**
	x	0.41	0.005	0.021	8E-04	0.249	0.06	0.017	0.426	0.069	N.D.	N.D.	N.D.	N.D.	N.D.	**Primary root 2-4 cm**
		x	0.249	0.467	0.179	0.624	0.348	0.256	0.698	0.483	N.D.	N.D.	N.D.	N.D.	N.D.	**Primary root 4-8 cm**
			x	0.556	0.063	0.035	0.003	0.008	0.698	0.027	N.D.	N.D.	N.D.	N.D.	N.D.	**Primary root 8-16 cm**
				x	0.05	0.048	0.015	0.009	0.448	0.013	N.D.	N.D.	N.D.	N.D.	N.D.	**Seminal root 1-4 cm**
					x	0.015	0.003	5E-04	0.171	0.042	N.D.	N.D.	N.D.	N.D.	N.D.	**Crown root 0.5-4 cm**
						x	0.022	0.018	0.706	0.201	N.D.	N.D.	N.D.	N.D.	N.D.	**Stele**
							x	0.136	0.257	0.263	N.D.	N.D.	N.D.	N.D.	N.D.	**Cortex**
								x	0.172	0.409	N.D.	N.D.	N.D.	N.D.	N.D.	**Elongation zone**
									x	0.29	N.D.	N.D.	N.D.	N.D.	N.D.	**Meristem**
										x	N.D.	N.D.	N.D.	N.D.	N.D.	**Lateral root**
											x	N.D.	N.D.	N.D.	N.D.	**Coleoptile (d)**
												x	N.D.	N.D.	N.D.	**Coleoptile (l)**
													x	N.D.	N.D.	**Mesocotyl**
														x	N.D.	**Coleopilar node**
															x	**Leaf**

(pink)	*p* ≤0.05
(orange)	*p* ≤0.01
(green)	*p* ≤0.001
N.D.	not detectable

ZmIAA14

Primary root 1-2 cm	Primary root 2-4 cm	Primary root 4-8 cm	Primary root 8-16 cm	Seminal root 1-4 cm	Crown root 0.5-4 cm	Stele	Cortex	Elongation zone	Meristem	Lateral root	Coleoptile (d)	Coleoptile (l)	Mesocotyl	Coleoptilar node	Leaf	
x	0.43	0.306	0.194	0.055	0.003	0.257	0.029	0.215	0.034	0.786	0.009	0.007	0.007	0.006	0.008	**Primary root 1-2 cm**
	x	0.706	0.081	0.242	6E-04	0.298	0.021	0.34	0.012	0.508	0.004	0.004	0.006	0.005	0.005	**Primary root 2-4 cm**
		x	0.017	0.279	0.001	0.304	0.023	0.24	0.008	0.354	7E-04	6E-04	0.001	0.001	0.001	**Primary root 4-8 cm**
			x	0.058	3E-04	0.194	0.019	0.688	0.008	0.965	3E-04	6E-05	1E-04	9E-05	3E-04	**Primary root 8-16 cm**
				x	0.148	0.631	0.052	0.051	0.037	0.521	0.019	0.015	0.018	0.017	0.019	**Seminal root 1-4 cm**
					x	0.947	0.102	0.006	5E-05	0.124	4E-05	9E-05	8E-05	6E-05	5E-05	**Crown root 0.5-4 cm**
						x	0.32	0.219	0.145	0.24	0.094	0.098	0.097	0.094	0.094	**Stele**
							x	0.033	0.016	0.305	0.012	0.011	0.013	0.012	0.012	**Cortex**
								x	0.154	0.512	0.029	0.027	0.024	0.022	0.026	**Elongation zone**
									x	0.654	0.001	0.003	0.002	9E-04	0.002	**Meristem**
										x	0.286	0.285	0.254	0.259	0.261	**Lateral root**
											x	0.636	0.343	0.62	0.606	**Coleoptile (d)**
												x	0.303	0.82	0.565	**Coleoptile (l)**
													x	0.034	0.277	**Mesocotyl**
														x	0.24	**Coleopilar node**
															x	**Leaf**

ZmIAA15

Primary root 1-2 cm	Primary root 2-4 cm	Primary root 4-8 cm	Primary root 8-16 cm	Seminal root 1-4 cm	Crown root 0.5-4 cm	Stele	Cortex	Elongation zone	Meristem	Lateral root	Coleoptile (d)	Coleoptile (l)	Mesocotyl	Coleoptilar node	Leaf	
x	0.366	0.486	0.042	0.169	0.027	0.738	0.199	0.075	0.119	0.216	0.007	0.019	0.063	0.069	0.023	**Primary root 1-2 cm**
	x	0.831	0.326	0.148	0.006	0.568	0.068	0.139	0.187	0.367	0.128	0.092	0.008	0.008	0.015	**Primary root 2-4 cm**
		x	0.08	0.096	0.009	0.594	0.124	0.242	0.204	0.231	0.053	0.016	0.151	0.108	0.014	**Primary root 4-8 cm**
			x	0.065	0.014	0.394	0.102	0.512	0.204	0.059	0.253	0.095	0.704	0.778	0.121	**Primary root 8-16 cm**
				x	0.836	0.627	0.852	0.104	0.122	0.78	0.065	0.059	0.099	0.09	0.057	**Seminal root 1-4 cm**
					x	0.475	0.446	0.006	0.021	0.707	0.009	0.007	0.006	0.004	0.004	**Crown root 0.5-4 cm**
						x	0.657	0.455	0.254	0.623	0.35	0.303	0.33	0.343	0.215	**Stele**
							x	0.072	0.088	0.818	0.074	0.065	0.042	0.043	0.038	**Cortex**
								x	0.328	0.217	0.126	0.07	0.096	0.062	0.008	**Elongation zone**
									x	0.011	0.983	0.742	0.96	0.883	0.166	**Meristem**
										x	0.096	0.086	0.226	0.227	0.119	**Lateral root**
											x	0.337	0.982	0.856	0.15	**Coleoptile (d)**
												x	0.774	0.57	0.145	**Coleoptile (l)**
													x	0.558	0.129	**Mesocotyl**
														x	0.032	**Coleopilar node**
															x	**Leaf**

ZmIAA16

Primary root 1-2 cm	Primary root 2-4 cm	Primary root 4-8 cm	Primary root 8-16 cm	Seminal root 1-4 cm	Crown root 0.5-4 cm	Stele	Cortex	Elongation zone	Meristem	Lateral root	Coleoptile (d)	Coleoptile (l)	Mesocotyl	Coleoptilar node	Leaf	
x	0.846	0.905	0.147	0.045	1E-04	0.284	0.007	0.041	0.018	0.208	0.045	0.02	0.014	0.102	0.031	**Primary root 1-2 cm**
	x	0.916	0.242	0.071	4E-04	0.318	0.004	0.111	0.033	0.369	0.072	0.038	0.027	0.209	0.072	**Primary root 2-4 cm**
		x	0.001	0.086	7E-04	0.254	0.003	0.021	8E-04	0.211	0.002	5E-04	3E-04	6E-04	4E-04	**Primary root 4-8 cm**
			x	0.051	6E-04	0.192	0.001	0.598	8E-04	0.188	0.005	8E-04	4E-04	0.611	0.005	**Primary root 8-16 cm**
				x	0.022	0.743	0.25	0.041	0.029	0.391	0.033	0.028	0.024	0.045	0.034	**Seminal root 1-4 cm**
					x	0.621	0.06	3E-04	4E-04	0.604	4E-04	4E-04	3E-04	4E-04	3E-04	**Crown root 0.5-4 cm**
						x	0.954	0.199	0.14	0.471	0.159	0.139	0.127	0.195	0.164	**Stele**
							x	0.003	9E-04	0.927	6E-04	7E-04	6E-04	0.001	0.001	**Cortex**
								x	0.012	0.205	0.181	0.029	0.014	0.634	0.103	**Elongation zone**
									x	0.177	0.072	0.982	0.034	0.005	0.007	**Meristem**
										x	0.186	0.173	0.165	0.189	0.182	**Lateral root**
											x	0.013	0.003	0.009	0.568	**Coleoptile (d)**
												x	6E-06	0.002	0.003	**Coleoptile (l)**
													x	7E-04	8E-04	**Mesocotyl**
														x	0.003	**Coleopilar node**
															x	**Leaf**

	p ≤0.05
	p ≤0.01
	p ≤0.001
N.D.	not detectable

ZmIAA17

Primary root 1-2 cm	Primary root 2-4 cm	Primary root 4-8 cm	Primary root 8-16 cm	Seminal root 1-4 cm	Crown root 0.5-4 cm	Stele	Cortex	Elongation zone	Meristem	Lateral root	Coleoptile (d)	Coleoptile (l)	Mesocotyl	Coleoptilar node	Leaf	
x	0.031	0.282	0.001	0.488	0.306	0.218	0.265	0.038	6E-04	0.066	6E-04	9E-04	0.093	0.002	0.074	**Primary root 1-2 cm**
	x	0.239	0.114	0.241	0.071	0.158	0.16	0.35	0.008	0.121	0.014	0.014	0.062	0.037	0.071	**Primary root 2-4 cm**
		x	0.081	0.337	0.063	0.185	0.16	0.213	0.025	0.332	0.03	0.043	0.115	0.077	0.982	**Primary root 4-8 cm**
			x	0.136	0.067	0.151	0.146	0.863	0.025	0.121	0.009	0.106	0.033	0.608	0.037	**Primary root 8-16 cm**
				x	0.901	0.404	0.47	0.088	0.103	0.203	0.106	0.123	0.647	0.132	0.349	**Seminal root 1-4 cm**
					x	0.294	0.248	0.084	0.041	0.311	0.045	0.05	0.7	0.06	0.148	**Crown root 0.5-4 cm**
						x	0.64	0.166	0.13	0.067	0.134	0.137	0.372	0.144	0.172	**Stele**
							x	0.159	0.121	0.423	0.124	0.131	0.637	0.142	0.203	**Cortex**
								x	0.225	0.308	0.27	0.451	0.061	0.687	0.156	**Elongation zone**
									x	0.432	0.038	0.002	0.025	0.008	0.005	**Meristem**
										x	0.647	0.143	0.265	0.021	0.053	**Lateral root**
											x	0.028	0.026	0.02	0.008	**Coleoptile (d)**
												x	0.029	0.046	0.007	**Coleoptile (l)**
													x	0.034	0.064	**Mesocotyl**
														x	0.012	**Coleopilar node**
															x	**Leaf**

ZmIAA18

Primary root 1-2 cm	Primary root 2-4 cm	Primary root 4-8 cm	Primary root 8-16 cm	Seminal root 1-4 cm	Crown root 0.5-4 cm	Stele	Cortex	Elongation zone	Meristem	Lateral root	Coleoptile (d)	Coleoptile (l)	Mesocotyl	Coleoptilar node	Leaf	
x	0.248	0.075	0.362	0.977	0.049	0.266	0.045	0.002	0.007	0.042	0.1	0.082	0.022	0.321	0.059	**Primary root 1-2 cm**
	x	0.083	0.12	0.55	0.034	0.283	0.04	0.005	0.009	0.2	0.103	0.084	0.023	0.593	0.087	**Primary root 2-4 cm**
		x	0.024	0.224	0.967	0.296	0.126	0.003	0.004	0.011	0.129	0.133	0.021	0.775	0.138	**Primary root 4-8 cm**
			x	0.714	0.018	0.256	0.045	1E-05	0.004	0.136	0.104	0.089	0.017	0.195	0.027	**Primary root 8-16 cm**
				x	0.153	0.263	0.071	0.043	0.049	0.497	0.106	0.098	0.021	0.388	0.07	**Seminal root 1-4 cm**
					x	0.303	0.085	0.002	0.002	0.1	0.117	0.109	0.024	0.845	0.16	**Crown root 0.5-4 cm**
						x	0.437	0.219	0.222	0.425	0.937	0.632	0.828	0.264	0.317	**Stele**
							x	0.019	0.022	0.345	0.098	0.06	0.098	0.235	0.428	**Cortex**
								x	0.132	0.119	0.088	0.065	0.013	0.037	0.008	**Elongation zone**
									x	0.261	0.091	0.069	0.012	0.042	0.007	**Meristem**
										x	0.337	0.295	0.119	0.29	0.096	**Lateral root**
											x	0.125	0.837	0.16	0.171	**Coleoptile (d)**
												x	0.418	0.198	0.236	**Coleoptile (l)**
													x	0.011	0.016	**Mesocotyl**
														x	0.092	**Coleopilar node**
															x	**Leaf**

ZmIAA19

Primary root 1-2 cm	Primary root 2-4 cm	Primary root 4-8 cm	Primary root 8-16 cm	Seminal root 1-4 cm	Crown root 0.5-4 cm	Stele	Cortex	Elongation zone	Meristem	Lateral root	Coleoptile (d)	Coleoptile (l)	Mesocotyl	Coleoptilar node	Leaf	
x	0.309	0.006	0.061	0.083	0.185	0.36	0.012	0.008	0.007	0.613	0.006	0.006	0.006	0.006	0.006	**Primary root 1-2 cm**
	x	0.256	0.68	0.21	0.119	0.447	0.028	0.007	0.007	0.34	0.007	0.007	0.008	0.007	0.007	**Primary root 2-4 cm**
		x	0.275	0.206	0.975	0.57	0.031	0.002	0.002	0.187	0.002	0.002	0.002	0.002	0.002	**Primary root 4-8 cm**
			x	0.197	0.511	0.451	0.031	0.001	0.002	0.095	0.001	0.001	0.001	0.001	0.001	**Primary root 8-16 cm**
				x	0.348	0.981	0.296	0.049	0.049	0.228	0.047	0.047	0.048	0.047	0.047	**Seminal root 1-4 cm**
					x	0.591	0.041	0.019	0.02	0.388	0.02	0.02	0.021	0.02	0.02	**Crown root 0.5-4 cm**
						x	0.716	0.201	0.199	0.484	0.195	0.195	0.199	0.195	0.195	**Stele**
							x	0.009	0.009	0.295	0.009	0.009	0.009	0.009	0.009	**Cortex**
								x	0.284	0.246	0.044	0.05	0.884	0.05	0.049	**Elongation zone**
									x	0.233	0.026	0.031	0.627	0.03	0.031	**Meristem**
										x	0.204	0.205	0.213	0.204	0.203	**Lateral root**
											x	0.008	0.001	0.003	0.107	**Coleoptile (d)**
												x	0.001	0.502	0.17	**Coleoptile (l)**
													x	0.002	0.001	**Mesocotyl**
														x	0.102	**Coleopilar node**
															x	**Leaf**

	$p \leq 0.05$
	$p \leq 0.01$
	$p \leq 0.001$
N.D.	not detectable

ZmIAA20

Primary root 1-2 cm	Primary root 2-4 cm	Primary root 4-8 cm	Primary root 8-16 cm	Seminal root 1-4 cm	Crown root 0.5-4 cm	Stele	Cortex	Elongation zone	Meristem	Lateral root	Coleoptile (d)	Coleoptile (l)	Mesocotyl	Coleoptilar node	Leaf	
x	0.067	0.007	0.009	0.215	0.039	0.083	0.038	0.112	0.201	0.412	6E-04	0.002	0.009	0.003	5E-04	**Primary root 1-2 cm**
	x	0.07	0.004	0.292	0.023	0.215	0.05	0.032	0.036	0.259	0.003	0.002	0.009	0.003	0.001	**Primary root 2-4 cm**
		x	0.133	0.456	0.356	0.746	0.007	0.009	0.01	0.081	5E-04	0.004	0.009	0.003	0.002	**Primary root 4-8 cm**
			x	0.829	0.002	0.414	0.008	0.007	0.01	0.137	0.125	0.003	0.009	0.003	0.015	**Primary root 8-16 cm**
				x	0.385	0.55	0.21	0.204	0.207	0.032	0.692	0.017	0.008	0.002	0.263	**Seminal root 1-4 cm**
					x	0.476	0.034	0.027	0.029	0.266	0.008	0.002	0.009	0.003	0.003	**Crown root 0.5-4 cm**
						x	0.074	0.07	0.07	0.457	0.063	0.007	0.01	0.006	1E-03	**Stele**
							x	0.335	0.597	0.028	6E-04	0.002	0.009	0.003	4E-04	**Cortex**
								x	0.125	0.007	8E-04	0.002	0.009	0.003	5E-04	**Elongation zone**
									x	0.195	8E-04	0.002	0.009	0.003	4E-04	**Meristem**
										x	0.02	0.047	0.093	0.149	0.086	**Lateral root**
											x	0.008	0.01	0.004	0.026	**Coleoptile (d)**
												x	0.01	0.006	0.033	**Coleoptile (l)**
													x	0.011	0.01	**Mesocotyl**
														x	0.008	**Coleopilar node**
															x	**Leaf**

ZmIAA21

Primary root 1-2 cm	Primary root 2-4 cm	Primary root 4-8 cm	Primary root 8-16 cm	Seminal root 1-4 cm	Crown root 0.5-4 cm	Stele	Cortex	Elongation zone	Meristem	Lateral root	Coleoptile (d)	Coleoptile (l)	Mesocotyl	Coleoptilar node	Leaf	
x	0.02	0.005	0.025	0.046	0.014	0.209	0.028	0.024	0.059	0.507	0.027	0.001	0.11	0.412	0.108	**Primary root 1-2 cm**
	x	0.748	0.65	0.359	0.6	0.277	0.056	0.005	0.007	0.193	0.035	0.033	0.215	0.079	0.741	**Primary root 2-4 cm**
		x	0.64	0.193	0.936	0.26	0.082	3E-04	5E-04	0.045	0.005	0.011	0.219	0.043	0.034	**Primary root 4-8 cm**
			x	0.389	0.914	0.249	0.075	5E-04	5E-04	0.081	0.009	0.018	0.212	0.014	0.052	**Primary root 8-16 cm**
				x	0.322	0.252	0.06	0.02	0.029	0.014	0.077	0.058	0.221	0.211	0.063	**Seminal root 1-4 cm**
					x	0.277	0.036	0.006	0.008	0.253	0.03	0.015	0.276	0.125	0.472	**Crown root 0.5-4 cm**
						x	0.504	0.169	0.172	0.384	0.254	0.258	0.379	0.228	0.436	**Stele**
							x	0.017	0.018	0.268	0.048	0.04	0.675	0.101	0.503	**Cortex**
								x	0.057	0.018	0.27	0.735	0.068	0.734	0.039	**Elongation zone**
									x	0.124	0.661	0.715	0.071	0.936	0.031	**Meristem**
										x	0.232	0.368	0.067	0.901	0.042	**Lateral root**
											x	0.765	0.078	0.644	0.062	**Coleoptile (d)**
												x	0.087	0.632	0.092	**Coleoptile (l)**
													x	0.012	0.089	**Mesocotyl**
														x	0.075	**Coleopilar node**
															x	**Leaf**

ZmIAA23

Primary root 1-2 cm	Primary root 2-4 cm	Primary root 4-8 cm	Primary root 8-16 cm	Seminal root 1-4 cm	Crown root 0.5-4 cm	Stele	Cortex	Elongation zone	Meristem	Lateral root	Coleoptile (d)	Coleoptile (l)	Mesocotyl	Coleoptilar node	Leaf	
x	0.075	0.013	0.052	0.224	0.052	0.28	0.008	0.031	0.111	0.826	0.047	0.036	0.054	0.066	0.249	**Primary root 1-2 cm**
	x	0.399	0.221	0.338	0.203	0.46	0.031	0.029	0.034	0.216	0.025	0.025	0.026	0.033	0.032	**Primary root 2-4 cm**
		x	0.159	0.322	0.698	0.356	0.016	9E-04	0.003	0.277	0.003	0.002	0.003	0.001	0.01	**Primary root 4-8 cm**
			x	0.879	0.87	0.323	0.016	4E-04	0.003	0.285	0.001	0.001	0.001	8E-05	0.002	**Primary root 8-16 cm**
				x	0.838	0.332	0.02	0.038	0.069	0.728	0.054	0.052	0.053	0.048	0.119	**Seminal root 1-4 cm**
					x	0.368	0.007	0.033	0.057	0.293	0.033	0.03	0.035	0.047	0.073	**Crown root 0.5-4 cm**
						x	0.656	0.226	0.23	0.5	0.231	0.231	0.234	0.228	0.239	**Stele**
							x	0.009	0.01	0.029	0.009	0.009	0.009	0.009	0.011	**Cortex**
								x	0.138	0.276	0.352	0.351	0.168	0.105	0.09	**Elongation zone**
									x	0.572	0.309	0.308	0.515	0.224	0.104	**Meristem**
										x	0.236	0.222	0.223	0.419	0.886	**Lateral root**
											x	0.679	0.172	0.644	0.088	**Coleoptile (d)**
												x	0.165	0.583	0.104	**Coleoptile (l)**
													x	0.981	0.157	**Mesocotyl**
														x	0.121	**Coleopilar node**
															x	**Leaf**

(pink)	*p* ≤0.05
(yellow)	*p* ≤0.01
(green)	*p* ≤0.001
N.D.	not detectable

ZmIAA25

Primary root 1-2 cm	Primary root 2-4 cm	Primary root 4-8 cm	Primary root 8-16 cm	Seminal root 1-4 cm	Crown root 0.5-4 cm	Stele	Cortex	Elongation zone	Meristem	Lateral root	Coleoptile (d)	Coleoptile (l)	Mesocotyl	Coleoptilar node	Leaf	
x	0.013	0.116	0.022	0.092	0.029	0.371	0.086	0.03	0.039	0.685	0.009	0.01	0.493	0.022	0.121	**Primary root 1-2 cm**
	x	0.007	9E-04	0.008	0.034	0.438	0.025	0.145	0.492	0.559	0.009	0.615	0.131	0.154	0.383	**Primary root 2-4 cm**
		x	0.543	0.314	0.154	0.064	0.183	0.004	9E-04	0.986	0.006	0.004	0.977	5E-04	0.005	**Primary root 4-8 cm**
			x	0.448	0.163	0.043	0.169	0.002	9E-04	0.874	4E-04	2E-04	0.782	0.002	0.004	**Primary root 8-16 cm**
				x	0.69	0.002	0.457	0.002	0.022	0.814	0.004	0.012	0.558	0.007	0.014	**Seminal root 1-4 cm**
					x	0.153	0.636	0.048	0.025	0.517	0.03	0.024	0.405	0.036	0.057	**Crown root 0.5-4 cm**
						x	0.035	0.1	0.357	0.812	0.142	0.419	0.194	0.166	0.718	**Stele**
							x	0.017	0.034	0.308	0.016	0.019	0.094	0.017	0.017	**Cortex**
								x	0.963	0.571	0.505	0.294	0.079	0.818	0.049	**Elongation zone**
									x	0.473	0.777	0.559	0.081	0.852	0.25	**Meristem**
										x	0.509	0.529	0.603	0.528	0.663	**Lateral root**
											x	0.076	0.087	0.741	0.074	**Coleoptile (d)**
												x	0.102	0.173	0.215	**Coleoptile (l)**
													x	0.061	0.094	**Mesocotyl**
														x	0.018	**Coleopilar node**
															x	**Leaf**

ZmIAA27

Primary root 1-2 cm	Primary root 2-4 cm	Primary root 4-8 cm	Primary root 8-16 cm	Seminal root 1-4 cm	Crown root 0.5-4 cm	Stele	Cortex	Elongation zone	Meristem	Lateral root	Coleoptile (d)	Coleoptile (l)	Mesocotyl	Coleoptilar node	Leaf	
x	0.681	0.276	0.129	0.463	0.01	0.264	0.206	0.018	0.021	0.852	0.005	0.005	0.006	0.006	0.008	**Primary root 1-2 cm**
	x	0.099	0.374	0.365	0.042	0.183	0.169	0.116	0.082	0.276	0.028	0.026	0.026	0.026	0.03	**Primary root 2-4 cm**
		x	0.057	0.482	0.033	0.334	0.2	0.012	0.013	0.412	0.009	0.008	0.009	0.009	0.01	**Primary root 4-8 cm**
			x	0.051	0.012	0.175	0.171	0.075	0.013	0.028	8E-04	8E-04	6E-04	8E-04	8E-04	**Primary root 8-16 cm**
				x	0.043	0.307	0.217	0.034	0.031	0.614	0.007	0.007	0.007	0.007	0.009	**Seminal root 1-4 cm**
					x	0.554	0.293	0.002	0.003	0.252	0.002	0.002	0.003	0.002	0.003	**Crown root 0.5-4 cm**
						x	0.55	0.138	0.13	0.247	0.108	0.105	0.105	0.106	0.11	**Stele**
							x	0.142	0.141	0.427	0.127	0.126	0.127	0.127	0.13	**Cortex**
								x	0.251	0.205	0.017	0.013	0.019	0.015	0.029	**Elongation zone**
									x	0.132	0.014	0.01	0.014	0.011	0.021	**Meristem**
										x	0.03	0.028	0.015	0.026	0.019	**Lateral root**
											x	0.078	0.584	0.143	0.024	**Coleoptile (d)**
												x	0.28	0.034	0.004	**Coleoptile (l)**
													x	0.481	8E-04	**Mesocotyl**
														x	0.003	**Coleopilar node**
															x	**Leaf**

ZmIAA28

Primary root 1-2 cm	Primary root 2-4 cm	Primary root 4-8 cm	Primary root 8-16 cm	Seminal root 1-4 cm	Crown root 0.5-4 cm	Stele	Cortex	Elongation zone	Meristem	Lateral root	Coleoptile (d)	Coleoptile (l)	Mesocotyl	Coleoptilar node	Leaf	
x	0.287	0.076	0.16	0.047	5E-04	0.225	0.066	0.03	0.445	0.809	0.175	0.463	0.328	0.146	0.691	**Primary root 1-2 cm**
	x	0.488	0.933	0.643	0.109	0.251	0.067	0.031	0.108	0.165	0.036	0.09	0.83	0.132	0.369	**Primary root 2-4 cm**
		x	0.527	0.905	0.103	0.266	0.163	0.001	0.014	0.236	0.002	0.002	0.191	0.014	0.023	**Primary root 4-8 cm**
			x	0.483	0.06	0.235	0.114	0.049	0.014	0.314	0.069	0.167	0.947	0.092	0.308	**Primary root 8-16 cm**
				x	0.05	0.29	0.194	0.014	0.1	0.317	0.054	0.062	0.518	0.032	0.142	**Seminal root 1-4 cm**
					x	0.337	0.272	0.001	0.02	0.02	0.008	0.005	0.095	0.001	0.02	**Crown root 0.5-4 cm**
						x	0.497	0.187	0.187	0.369	0.187	0.21	0.227	0.209	0.215	**Stele**
							x	0.043	0.07	0.311	0.051	0.062	0.122	0.061	0.098	**Cortex**
								x	0.201	0.126	0.216	0.003	0.024	0.012	0.019	**Elongation zone**
									x	0.975	0.235	0.607	0.003	0.756	0.04	**Meristem**
										x	0.59	0.737	0.369	0.553	0.724	**Lateral root**
											x	0.056	0.006	0.223	0.017	**Coleoptile (d)**
												x	0.063	0.784	0.15	**Coleoptile (l)**
													x	0.106	0.149	**Mesocotyl**
														x	0.134	**Coleopilar node**
															x	**Leaf**

(pink)	$p \leq 0.05$
(orange)	$p \leq 0.01$
(green)	$p \leq 0.001$
N.D.	not detectable

ZmIAA29

Primary root 1-2 cm	Primary root 2-4 cm	Primary root 4-8 cm	Primary root 8-16 cm	Seminal root 1-4 cm	Crown root 0.5-4 cm	Stele	Cortex	Elongation zone	Meristem	Lateral root	Coleoptile (d)	Coleoptile (l)	Mesocotyl	Coleoptilar node	Leaf	
x	0.29	0.114	0.829	0.154	0.099	0.439	0.022	0.005	0.066	0.646	0.007	0.005	0.125	0.024	0.023	**Primary root 1-2 cm**
	x	0.815	0.289	0.535	0.05	0.51	0.034	0.006	0.026	0.221	0.013	0.007	0.094	0.024	0.016	**Primary root 2-4 cm**
		x	0.375	0.288	0.254	0.486	0.034	0.002	0.011	0.122	0.002	0.002	0.011	0.003	0.005	**Primary root 4-8 cm**
			x	0.295	0.102	0.406	0.034	0.001	0.011	0.157	0.002	0.001	0.035	0.003	0.001	**Primary root 8-16 cm**
				x	0.937	0.711	0.036	0.039	0.1	0.084	0.041	0.041	0.098	0.062	0.065	**Seminal root 1-4 cm**
					x	0.708	0.041	0.012	0.031	0.263	0.016	0.011	0.062	0.027	0.021	**Crown root 0.5-4 cm**
						x	0.416	0.215	0.291	0.502	0.23	0.221	0.323	0.259	0.252	**Stele**
							x	0.016	0.024	0.341	0.017	0.016	0.025	0.021	0.02	**Cortex**
								x	0.001	0.012	0.075	0.051	0.003	0.005	0.003	**Elongation zone**
									x	0.321	0.01	0.002	0.222	0.035	0.007	**Meristem**
										x	0.05	0.006	0.59	0.181	0.139	**Lateral root**
											x	0.157	0.001	0.007	0.021	**Coleoptile (d)**
												x	0.005	0.01	0.007	**Coleoptile (l)**
													x	0.004	0.011	**Mesocotyl**
														x	0.232	**Coleopilar node**
															x	**Leaf**

ZmIAA30

Primary root 1-2 cm	Primary root 2-4 cm	Primary root 4-8 cm	Primary root 8-16 cm	Seminal root 1-4 cm	Crown root 0.5-4 cm	Stele	Cortex	Elongation zone	Meristem	Lateral root	Coleoptile (d)	Coleoptile (l)	Mesocotyl	Coleoptilar node	Leaf	
x	0.099	0.656	0.07	0.04	0.001	0.086	0.732	0.07	0.087	0.169	N.D.	N.D.	N.D.	N.D.	N.D.	**Primary root 1-2 cm**
	x	0.31	0.421	0.028	4E-04	0.592	0.29	3E-04	0.023	0.379	N.D.	N.D.	N.D.	N.D.	N.D.	**Primary root 2-4 cm**
		x	0.313	0.254	0.01	0.418	0.554	0.208	0.231	0.451	N.D.	N.D.	N.D.	N.D.	N.D.	**Primary root 4-8 cm**
			x	0.02	3E-04	0.207	0.289	0.143	0.231	0.326	N.D.	N.D.	N.D.	N.D.	N.D.	**Primary root 8-16 cm**
				x	8E-04	0.028	0.274	0.022	0.026	0.108	N.D.	N.D.	N.D.	N.D.	N.D.	**Seminal root 1-4 cm**
					x	6E-04	0.011	4E-04	5E-04	0.022	N.D.	N.D.	N.D.	N.D.	N.D.	**Crown root 0.5-4 cm**
						x	0.381	0.134	0.221	0.722	N.D.	N.D.	N.D.	N.D.	N.D.	**Stele**
							x	0.203	0.226	0.529	N.D.	N.D.	N.D.	N.D.	N.D.	**Cortex**
								x	0.163	0.18	N.D.	N.D.	N.D.	N.D.	N.D.	**Elongation zone**
									x	0.134	N.D.	N.D.	N.D.	N.D.	N.D.	**Meristem**
										x	N.D.	N.D.	N.D.	N.D.	N.D.	**Lateral root**
											x	N.D.	N.D.	N.D.	N.D.	**Coleoptile (d)**
												x	N.D.	N.D.	N.D.	**Coleoptile (l)**
													x	N.D.	N.D.	**Mesocotyl**
														x	N.D.	**Coleopilar node**
															x	**Leaf**

ZmIAA32

Primary root 1-2 cm	Primary root 2-4 cm	Primary root 4-8 cm	Primary root 8-16 cm	Seminal root 1-4 cm	Crown root 0.5-4 cm	Stele	Cortex	Elongation zone	Meristem	Lateral root	Coleoptile (d)	Coleoptile (l)	Mesocotyl	Coleoptilar node	Leaf	
x	0.028	0.163	0.031	0.061	0.075	0.143	0.06	0.009	0.007	0.17	0.004	0.004	0.004	0.004	0.004	**Primary root 1-2 cm**
	x	0.759	0.506	0.599	0.438	0.722	0.077	0.005	0.019	0.074	0.001	0.001	0.002	0.001	0.001	**Primary root 2-4 cm**
		x	0.386	0.851	0.103	0.99	0.144	0.003	0.028	0.077	0.002	0.002	0.002	0.002	0.002	**Primary root 4-8 cm**
			x	0.231	0.836	0.289	0.069	0.003	0.028	0.005	0.002	0.002	0.002	0.002	0.002	**Primary root 8-16 cm**
				x	0.377	0.862	0.025	0.009	0.003	0.162	0.003	0.003	0.003	0.002	0.002	**Seminal root 1-4 cm**
					x	0.057	0.32	0.002	0.069	0.122	0.002	0.003	0.002	0.002	0.002	**Crown root 0.5-4 cm**
						x	0.142	0.002	0.026	0.13	0.002	0.003	0.002	0.002	0.002	**Stele**
							x	0.036	0.027	0.151	0.002	0.002	0.003	0.002	0.002	**Cortex**
								x	0.076	0.157	0.005	0.009	0.007	0.004	0.004	**Elongation zone**
									x	0.246	0.005	0.006	0.006	0.005	0.005	**Meristem**
										x	0.201	0.256	0.233	0.187	0.189	**Lateral root**
											x	0.237	0.255	0.005	0.008	**Coleoptile (d)**
												x	0.013	0.002	0.005	**Coleoptile (l)**
													x	0.095	0.226	**Mesocotyl**
														x	0.034	**Coleopilar node**
															x	**Leaf**

	p ≤0.05
	p ≤0.01
	p ≤0.001
N.D.	not detectable

ZmIAA33

Primary root 1-2 cm	Primary root 2-4 cm	Primary root 4-8 cm	Primary root 8-16 cm	Seminal root 1-4 cm	Crown root 0.5-4 cm	Stele	Cortex	Elongation zone	Meristem	Lateral root	Coleoptile (d)	Coleoptile (l)	Mesocotyl	Coleoptilar node	Leaf	
x	0.066	0.006	0.002	0.143	0.005	0.139	0.14	0.326	0.305	0.181	0.137	0.127	0.003	0.934	0.142	**Primary root 1-2 cm**
	x	0.024	9E-04	0.188	6E-05	0.171	0.435	0.024	0.006	0.608	0.003	0.003	0.03	0.015	0.004	**Primary root 2-4 cm**
		x	0.008	0.346	0.264	0.311	0.124	0.037	0.009	0.354	0.007	0.006	0.557	0.009	0.007	**Primary root 4-8 cm**
			x	0.791	0.003	0.62	0.016	0.008	0.009	0.117	0.001	0.001	0.035	0.001	0.001	**Primary root 8-16 cm**
				x	0.271	0.862	0.289	0.032	0.134	0.149	0.129	0.129	0.386	0.146	0.13	**Seminal root 1-4 cm**
					x	0.239	0.672	0.005	5E-04	0.18	3E-04	3E-04	0.203	9E-04	3E-04	**Crown root 0.5-4 cm**
						x	0.263	0.194	0.123	0.425	0.121	0.121	0.301	0.134	0.121	**Stele**
							x	0.177	0.14	0.766	0.115	0.113	0.164	0.17	0.116	**Cortex**
								x	0.137	0.131	0.314	0.242	0.024	0.015	0.92	**Elongation zone**
									x	0.097	0.05	0.04	0.006	0.013	0.048	**Meristem**
										x	0.136	0.131	0.049	0.187	0.128	**Lateral root**
											x	0.092	0.005	0.001	0.288	**Coleoptile (d)**
												x	0.005	7E-04	0.024	**Coleoptile (l)**
													x	0.006	0.005	**Mesocotyl**
														x	7E-04	**Coleopilar node**
															x	**Leaf**

ZmIAA34

Primary root 1-2 cm	Primary root 2-4 cm	Primary root 4-8 cm	Primary root 8-16 cm	Seminal root 1-4 cm	Crown root 0.5-4 cm	Stele	Cortex	Elongation zone	Meristem	Lateral root	Coleoptile (d)	Coleoptile (l)	Mesocotyl	Coleoptilar node	Leaf	
x	0.985	0.868	0.577	0.233	0.148	0.646	0.016	0.008	0.043	0.028	0.007	0.008	0.007	0.007	0.007	**Primary root 1-2 cm**
	x	0.9	0.425	0.4	0.019	0.632	0.004	0.019	0.008	0.077	0.002	0.016	0.002	0.003	0.002	**Primary root 2-4 cm**
		x	0.23	0.23	0.073	0.62	7E-04	0.005	0.02	0.046	1E-04	0.002	1E-04	8E-05	9E-05	**Primary root 4-8 cm**
			x	0.175	0.038	0.573	0.002	0.033	0.02	0.034	6E-04	0.002	6E-04	8E-04	5E-04	**Primary root 8-16 cm**
				x	0.573	0.835	0.031	0.042	0.081	0.013	0.016	0.074	0.016	0.016	0.016	**Seminal root 1-4 cm**
					x	0.959	0.005	0.012	0.011	0.153	0.004	0.02	0.004	0.004	0.004	**Crown root 0.5-4 cm**
						x	0.345	0.395	0.407	0.503	0.278	0.374	0.279	0.292	0.28	**Stele**
							x	0.157	0.093	0.084	4E-04	0.006	2E-04	0.002	9E-04	**Cortex**
								x	0.426	0.126	0.014	0.009	0.014	0.018	0.017	**Elongation zone**
									x	0.089	0.017	0.065	0.016	0.025	0.019	**Meristem**
										x	0.017	0.017	0.025	0.002	0.075	**Lateral root**
											x	0.003	0.303	0.01	0.032	**Coleoptile (d)**
												x	0.076	0.028	0.04	**Coleoptile (l)**
													x	0.013	0.239	**Mesocotyl**
														x	0.027	**Coleopilar node**
															x	**Leaf**

Figure S4
